# A Review on the Fate of Legacy and Alternative Antimicrobials and Their Metabolites during Wastewater and Sludge Treatment

**DOI:** 10.3390/ijms21239241

**Published:** 2020-12-03

**Authors:** Timothy Abbott, Gokce Kor-Bicakci, Mohammad S. Islam, Cigdem Eskicioglu

**Affiliations:** 1 UBC Bioreactor Technology Group, School of Engineering, University of British Columbia Okanagan Campus, Kelowna, BC V1V 1V7, Canada; tim.abbott@alumni.ubc.ca (T.A.); gokce.kor@gmail.com (G.K.-B.); imshowkat69@gmail.com (M.S.I.); 2 Institute of Environmental Sciences, Bogazici University, Bebek, 34342 Istanbul, Turkey

**Keywords:** antimicrobials, antimicrobial metabolites, transformation products, triclosan, triclocarban, emerging alternative antimicrobials, quaternary ammonium compounds, benzalkonium chlorides, wastewater treatment, sludge digestion

## Abstract

Antimicrobial compounds are used in a broad range of personal care, consumer and healthcare products and are frequently encountered in modern life. The use of these compounds is being reexamined as their safety, effectiveness and necessity are increasingly being questioned by regulators and consumers alike. Wastewater often contains significant amounts of these chemicals, much of which ends up being released into the environment as existing wastewater and sludge treatment processes are simply not designed to treat many of these contaminants. Furthermore, many biotic and abiotic processes during wastewater treatment can generate significant quantities of potentially toxic and persistent antimicrobial metabolites and byproducts, many of which may be even more concerning than their parent antimicrobials. This review article explores the occurrence and fate of two of the most common legacy antimicrobials, triclosan and triclocarban, their metabolites/byproducts during wastewater and sludge treatment and their potential impacts on the environment. This article also explores the fate and transformation of emerging alternative antimicrobials and addresses some of the growing concerns regarding these compounds. This is becoming increasingly important as consumers and regulators alike shift away from legacy antimicrobials to alternative chemicals which may have similar environmental and human health concerns.

## 1. Introduction

### 1.1. Introduction to Emerging Contaminants

Emerging contaminants consist of wide-ranging and expanding classes of anthropogenic and naturally occurring unregulated substances, on the occurrence, behavior, environmental fate and toxicity of which there are limited data [1,2,3]. They include pharmaceutical and personal care products (PPCPs), steroid hormones, brominated flame retardants, surfactants, pesticides, herbicides, perfluorinated compounds, nanoparticles and many other emerging compounds that are commonly found in municipal, agricultural and industrial wastewater [1,2,3,4,5]. These chemicals are potentially suspected to have adverse effects on aquatic environments, terrestrial organisms and humans [6]. Wastewater treatment plants (WWTPs) are the major transfer route for emerging contaminants to be released into the environment, in addition to many other minor routes [4,5]. Municipal WWTPs are not capable of fully treating these pollutants, as current facilities are generally designed to remove readily/moderately biodegradable carbon, nitrogen and phosphorus compounds and microorganisms [7]. While many emerging contaminants are metabolized and degraded to varying degrees during biological, chemical and physical processes, their metabolites/byproducts and unaltered parent compounds remain in the treated effluents or accumulate in wastewater treatment sludge. Emerging contaminants have been detected in concentrations ranging from a few ng/L to several μg/L in wastewater, surface water, groundwater and drinking water, while their concentrations have been reported up to μg/g dry weight (dw) in sludge, biosolids, biosolid-amended agricultural soils and sediment samples [2,8,9].

Depending on the physicochemical properties of the compounds and treatment technology used at the WWTP, wastewater treatment sludge is the endpoint of many hydrophobic and lipophilic compounds by sorption [10,11]. The presence of these chemicals in sludge hampers the beneficial reuse of biosolids in land application [12]. Many of them can be taken up by plants and terrestrial organisms, transferred into groundwater and/or accumulate in the food chain [13,14,15]. For this reason, understanding the occurrence, behavior and fate of persistent compounds in sludge has become an emerging concern for many researchers and regulators. A large number of studies have been conducted regarding the fate of persistent organic contaminants, such as polycyclic aromatic hydrocarbons, chlorinated dioxins/furans and polychlorinated biphenyls in environmental matrices over the past thirty years. The concentrations of these traditional contaminants in sludge have declined significantly with effective source control [12,16,17]. Currently, there is a clear trend towards understanding the behavior and fate of unregulated emerging contaminants, such as PPCPs, in solid matrices and their potential risks to humans and wildlife that has emerged during the last decade [9,15,18,19].

Pharmaceutical and personal care products are a class of emerging contaminants that are produced and consumed at high volumes all over the world. PPCPs include a large group of prescription and over-the-counter medications that are widely used in human medicine, veterinary drugs, in animal husbandry as growth promoters and as active and inert ingredients for personal care purposes [20,21]. PPCPs can be classified into many categories depending on their applications and commercial availability: analgesics, antibiotics, antifungals, anticoagulants, psychiatric drugs, hormones, lipid regulators, beta-blockers, stimulants, fragrances, antiseptics, ultraviolet (UV) filters, preservatives, insect repellents, non-ionic surfactants, and so on [20,21,22,23,24]. In spite of many potential benefits of the different classes of PPCPs to society, extensive use of these chemicals has led to their occurrence in various environmental compartments, including sludge, biosolids, agricultural soils and sediments as well as surface water, groundwater and drinking water [2,4,20,23,25,26,27,28,29,30].

**Figure 1:**
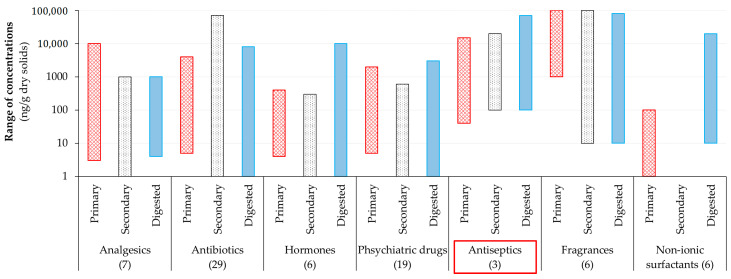
Occurrence of different classes of pharmaceuticals and personal care products in primary, secondary and digested sludges (the number of compounds for each class is shown in brackets). Adapted from reference [23].

A review by Verlicchi and Zambello [23], based on 59 papers published between 2002 and 2015 referring to about 450 treatment facilities operating in 24 different countries, provided data regarding sludge concentrations for 152 pharmaceutical and 17 personal care products. In their study, the principally investigated classes of PPCPs were (1) analgesics (acetaminophen, diclofenac, ibuprofen, ketoprofen, mefenamic acid, naproxen and salicylic acid), (2) antibiotics (azithromycin, ciprofloxacin, clarithromycin, difloxacin, doxycycline, enrofloxacin, erythromycin, fleroxacin, gatifloxacin, josamycin, levofloxacin, lincomycin, lomefloxacin, moxifloxacin, norfloxacin, ofloxacin, oxytetracycline, roxithromycin, sarafloxacin, sparfloxacin, spiramycin, sulfadiazine, sulfamerazin, sulfamethazine, sulfamethoxazole, sulfapyridine, tetracycline, trimethoprim and tylosin), (3) hormones (diethylstibestrol, estradiol, estriol, estrone, ethinylestradiol and progesterone), (4) psychiatric drugs (amitriptyline, carbamazepine, citalopram, diazepam, desmethylfluvoxamine, desmethylmitrazepine, desmethylsertraline, escitalopram, fluoxetine, fluvoxamine, lamotrigine, lorazepam, mitrazapine, norfluoxetine, nortriptyline, o-desmethylvenlafaxine, paroxetine, sertraline and venlafaxine), (5) antiseptics (pipemic acid, triclosan (TCS) and triclocarban (TCC)), (6) fragrances (cashmeran, celestolide, galaxolide, phantolide, tonalide and traseolide) and (7) non-ionic surfactants (nonylphenol, nonylphenol mono ethoxylate, nonylphenol di ethoxylate, 4-tert-octylphenol, 4-tert-octylphenol mono ethoxylate and 4-tert-octylphenol di ethoxylate). The ranges of observed concentrations for these PPCPs in primary, secondary and digested (aerobic or anaerobic digestion) sludges are shown in Figure 1. The highest sludge concentrations were found for antiseptics, fragrances and antibiotics. Based on an environmental risk assessment, TCS and TCC were among the most critical compounds found in digested sludge and sludge-amended soil [23].

Increasing concerns have been raised regarding endocrine-disrupting antimicrobials because of their potential human and environmental health threats and their physicochemical characteristics [31]. Antimicrobial compounds, which are either natural or semi/synthetic in origin, kill or inhibit many harmful organisms [32]. A wide range of synthetic antimicrobial agents are used in pharmaceuticals, building materials, personal care products and household consumables [29]. Specifically, TCS and TCC are commonly used antimicrobial agents with fungicidal and bactericidal properties for killing microorganisms indiscriminately, rapidly and by non-specific action [31,33]. Alternative compounds including quaternary ammoniums and other antimicrobials are becoming increasingly common due to shifting consumer preferences and bans on TCS and TCC in Europe, the U.S. and elsewhere. Little is known regarding the presence, concentrations, fate and metabolites of these alternative compounds, which may present similar challenges to the legacy antimicrobials TCS and TCC.

**Figure 2:**
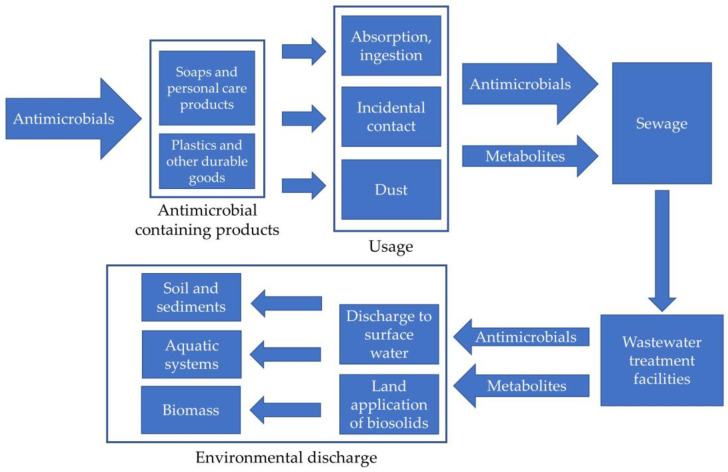
Flow of antimicrobials and their metabolites from products to the environment.

Even when used as they are intended, significant quantities of antimicrobials end up in wastewater, which are conveyed to wastewater treatment facilities in sewage [31]. Antimicrobials in fabrics, plastics and other durable goods along with those in soaps, cosmetics, mouthwashes and other products intended for direct contact with skin or mucosa can enter wastewater. Ingested or absorbed antimicrobials can be eliminated in humans and animals as their untransformed parent compounds, or they can be transformed into a variety of conjugated forms and/or into secondary metabolites [34,35,36,37,38]. Figure 2 represents the potential pathways for releases of legacy and emerging alternative antimicrobials and their metabolites from products to the environment.

### 1.2. Importance of Identification of Antimicrobials during Wastewater Treatment

The fate of antimicrobials during wastewater and sludge treatment can be determined by investigating their concentrations in both aqueous (i.e., wastewater) and solid (i.e., wastewater treatment sludge) matrices. Significant antimicrobial removals from the aqueous phase have been observed during wastewater treatment. For example, TCS removals of 91 to 98% have been reported during conventional activated sludge treatments [8,39,40,41,42], resulting in low TCS concentrations (0.11 to 10.7 µg/L) in treated effluent discharged to surface waters [8,39,40,41,42]. Similar results have been reported for TCC, which has similar chemical properties and usage patterns [23]. Despite apparent antimicrobial removals in the aqueous phase during wastewater treatment, only a portion of the antimicrobial removals reported in the literature are a result of biodegradation or mineralization. Many antimicrobials present in wastewater preferentially partition into particulate organic matter/sludge during wastewater treatment [8,31,39,42,43,44] due to their low water solubilities and high (≥4) logarithmic octanol–water partitioning coefficients (Log K_ow_). This typically results in substantially higher antimicrobial concentrations in wastewater sludge versus wastewater influent. Furthermore, many sludge stabilization treatments, including anaerobic digestion, degrade organic matter at a significantly higher rate than that of antimicrobials, resulting in higher antimicrobial concentrations in digested versus undigested sludge (in terms of mass analyte per gram of dry solids). Contaminants remaining in stabilized sludge may then enter the environment through land application of biosolids through soils [41,45,46,47], uptake in plants and crops [48,49,50,51,52,53] and movement through run-off or through groundwater [54,55].

A significant fraction of antimicrobials removed during wastewater treatment and/or sludge stabilization are often not completely mineralized [42,56]. Rather, they often form various conjugates and intermediate microbial metabolites, some of which be converted back into their parent compounds while others are even more toxic and biopersistant than their parent antimicrobials [7,57,58]. Once released to the environment, some of these byproducts can be transformed back into their parent antimicrobials, can accumulate in various environmental compartments or they can be further degraded. Therefore, it is vital that any examination of the fate of antimicrobials in wastewater and sludge treatments also considers the fate of these compounds and their partial degradation products.

## 2. Legacy Antimicrobial Triclosan

### 2.1. Background

**Figure 3:**
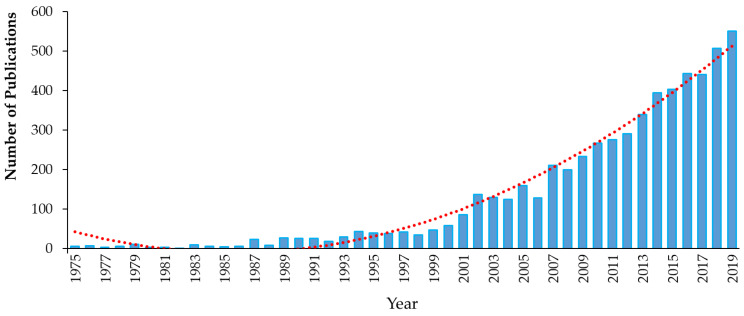
Number of scientific documents on the topic of “triclosan” published for the time period from 1975 to 2019 (data generated from reference [74]).

Triclosan (5-chloro-2-(2,4-dichlorophenoxy)phenol), chemical abstract service (CAS) #3380-34-5, is incorporated into numerous consumer and healthcare products in many countries around the world. TCS is a synthetic, nonionic, lipid-soluble broad-spectrum antimicrobial which is effective against most Gram-negative and Gram-positive bacteria [59,60], against some fungal species [61,62] and against some parasites and protozoa [63]. At low concentrations (≥0.1 mg/L), it shows bacteriostatic activity, while at higher concentrations (≥2.0 mg/L), it is bactericidal against many species, including some staphylococci, streptococci, mycobacteria, *Escherichia coli* (*E. coli*) and methicillin-resistant *Staphylococcus aureus* (MRSA) [63,64,65]. TCS rapidly permeates cell walls and disrupts multiple cellular processes, including ribonucleic acid synthesis and the production of macromolecules, and can prevent fatty acid synthesis [64,66,67]. Since its development in 1962 and its initial use in underarm deodorants and deodorizing soaps, the use of TCS has increased exponentially [68,69]. For example, the amount of TCS used in Europe increased from 350 tons per year in 1998 [70] to over 450 in 2006 [71]. However, little recent information on TCS production and use is available. TCS has been used in a wide range of products and applications, ranging from cosmetics, toothpastes, deodorants, tissues, hand and laundry soaps, shower gels and hand lotions/creams to pre-operative cleansers, surgical sutures, antiseptics and antiseptic wipes and medical devices [31,38,59,60,68,71,72,73]. TCS has also been used in a range of durable consumer products, ranging from sports equipment, fabrics, plastics, paints and coatings to packaging and product treatments (ibid). More than 5800 scientific papers have been published on the topic of “triclosan” between 1975 and 2019 (Figure 3).

As shown in Table 1, TCS is a halogenated biphenyl ether which has a similar structure and properties to several potent toxins, including dioxins, polychlorinated biphenyls and other persistent environmental contaminants [31]. TCS is highly lipophilic as it has a very high (≥4) Log K_ow_ value, is highly insoluble in water and is relatively non-volatile [75,76]. Due to its incomplete removal during wastewater treatment and sludge stabilization processes, TCS is consistently found throughout the environment in waters, soils, sediments and biota which receive treated wastewater effluent and/or stabilized wastewater residuals [5,31,39,42,43,77,78,79].

### 2.2. Transformation/Degradation Products and Metabolites of Triclosan

**Figure 4:**
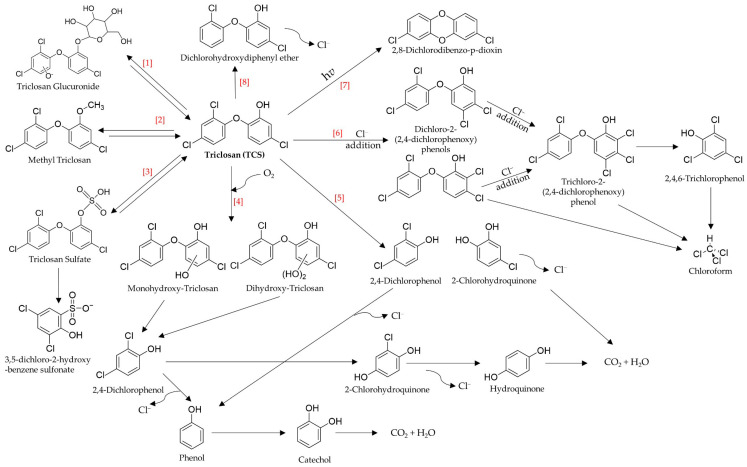
Common pathways for the transformation and degradation of triclosan. The primary reactions above are identified as follows: [1] glucuronidation; [2] methylation; [3] sulfation; [4] hydroxylation; [5] *meta*-cleavage of C-O bond; [6] chlorine substitution; [7] photolysis; [8] reductive dechlorination.

Triclosan can be transformed into various other compounds by biotic and abiotic processes and mechanisms within organisms, WWTPs and/or the environment. These can include various conjugated forms, photolysis products, chlorinated derivatives and partial mineralization metabolites, as shown in Figure 4. Some commonly detected TCS-related compounds are the TCS conjugates methyl triclosan (MeTCS), glucuronidated triclosan (TCS-gluc) and triclosan sulfate (TCS-SO_4_), several of which can have been shown to revert back into TCS [46,80,81]. About five percent of TCS is transformed into MeTCS through biomethylation of the TCS hydroxyl group during aerobic and anoxic processes in WWTPs [40,70]. The conjugate TCS-gluc (Figure 4) has been shown to be produced within organisms and has been found in animal or human blood, liver and intestines [46,82,83]. Chen et al. [57] observed that TCS was transformed into TCS-SO_4_ through sulfation during a batch simulated aerobic activated sludge process. This agrees with a later batch study by Trenholm et al. [84], who observed the rapid formation of TCS-SO_4_ under aerobic conditions and slower formation of TCS-SO_4_ under anoxic conditions using biological nutrient removal (BNR) sludge. Although Chen et al. [57] did not observe any decrease in TCS-SO_4_ once it was formed over 10 days, Trenholm et al. [84] observed that TCS-SO_4_ levels began to decrease after 5 days under aerobic conditions and observed the formation of a secondary TCS-SO_4_ metabolite, whose mass matched 3,5-dichloro-2-hydroxy-benzene sulfonate (or a closely related isomer thereof). TCS conjugates can also have significantly different physicochemical properties that those of its parent antimicrobial. For example, MeTCS is more persistent and bioaccumulative due to its even higher Log K_ow_ walue (5.0–5.1) and lower water solubility (0.4 mg/L) than those of TCS [46,85]. However, both TCS-SO_4_ and TCS-gluc are less lipophilic (estimated Log K_ow_ values of 2.18 and 2.82, respectively), are far more polar and are more soluble in water (65.17 and 11.58 mg/L at 25 °C, respectively) [82,86].

Triclosan can also form various intermediate metabolites, as shown in Figure 4, as many biotic processes have been shown to degrade it. Microalgae and many isolated pure-culture bacterial strains have been shown to partially or completely mineralize TCS. *Chlorella pyrenoidosa, Desmodesmus* sp. and *Scenedesmus* sp. have been shown to remove between 77.2 to 99.7% of TCS through reductive dechlorination, ether bond cleavage, hydroxylation or the production of TCS conjugates through methylation and/or glucosylation [87,88]. Hay et al. [89] observed partial TCS mineralization by an auxotrophic *Sphingomonas*-like strain isolated from an activated sludge basin. They observed the partial mineralization of the dichloro ring, with approximately 35% of ^14^C-TCS-labelled carbon being transformed to ^14^CO_2_ in a complex medium. The *Sphingomonas* sp. strain YL-JM2C, isolated from activated sludge, was shown to completely mineralize up to 99% of TCS within 72 h at an optimum pH of 7.0 and temperature of 30 °C and formed 2,4-dichlorophenol (2,4-DCP), 2-chlorohydroquinone and hydroquinone as intermediate decomposition products [90]. Mulla et al. [91] proposed that YL-JM2C first oxidatively transformed TCS into 2,4-DCP by breaking the ether bond between the two aromatic rings, likely facilitated by the enzymes monooxygenase and/or dioxygenase. Then, 2,4-DCP was reductively dechlorinated into 2-chlorohydroquinone and hydroquinone, before being fully mineralized into carbon dioxide. *Sphingomonas* sp. PH-07, originally isolated from activated sludge by Kim et al. [92], was shown to catabolize TCS into various hydroxylated metabolites, including monohydroxy-TCS and dihydroxy-TCS, and also formed 4-chlorophenol and 2,4-DCP, suggesting both aromatic rings underwent dihydroxylation [93]. Similar pathways were observed in an isolated *Sphingopyxis* strain KCY1, which was shown to rapidly degrade up to 90% of TCS within 24 h and showed 100% removal within 48 h [94]. Two oxygenase-expressing bacteria *Mycobacterium vaccae* JOB5 and *Rhodococcus jostii* RHA1 were able to degrade TCS through a *meta*-cleavage pathway when grown on propane and biphenyl, respectively [95]. Biphenyl-grown RHA1 produced 2,4-DCP, mono and dihydroxy-TCS and 2-chlorohydroquinone (ibid).

Mixed microbial cultures under methanogenic, denitrifying (anoxic) and aerobic conditions have also been shown to be effective in degrading TCS. For example, Gangadharan et al. [96] observed TCS removals of 87% and 80% during a 10-day solids retention time (SRT) when acetate was used as a co-substrate under methanogenic conditions and under denitrifying conditions, respectively, forming 2,4,DCP, phenol, catechol and other unidentified products under both conditions. Chen et al. [57] observed the formation of 2,4-DCP and 4-chlorocatechol (4-CC) in a 10-day batch simulated activated sludge reactor spiked with high levels of TCS which was formed through the *meta*-cleavage of the C-O bond between the two phenol rings. They also observed the production of monohydroxy-triclosan and dihydroxy-triclosan through the partial hydroxylation of the OH-containing TCS ring.

Other TCS transformation routes include abiotic processes, including chemical and photochemical reactions. However, many of these degradation products are far more toxic and biopersistant than the parent TCS. For example, Noutsopoulos et al. [97] found 92% TCS removal at a free chlorine dose of 40 mg Cl_2_/L and at a contact time of less than 5 min, but observed a 50- to 100-fold increase in *Vibrio fischeri* toxicity compared to TCS prior to chlorination. Multiple TCS-containing personal care products have also been shown to react with free chlorine to form higher-chlorinated TCS intermediates, including dichloro-2-(2,3-dichlorohenoxy)phenol and trichloro-2-(2,3-dichlorohenoxy)phenol. This was through Cl^−^ substitutions, various chlorophenols (2,4-DCP and 2,4,6-trichlorophenol) through the *meta*-cleavage of the TCS C-O bond or its higher-chlorinated intermediates and chloroform formation from ring cleavage of TCS, TCS-intermediates, chlorophenols and Cl^−^ substitutions [98,99,100]. The formation of even higher-chlorinated tetrachloro-2-(2,3-dichlorohenoxy)phenol and tetrachloro- and pentachloro-hydroxyl-TCS derivates was observed by Kanetoshi et al. [101] when TCS-impregnated fabric was exposed to a 0.2% sodium hypochlorite solution in warm water (45 °C), similar to what would be used for household laundry. TCS is particularly photodegradable in its phenolate form, and photochemical processes can transform a significant amount of TCS in aquatic systems, accounting for up to 80% of TCS removal in lakes [102,103]. Other studies have also shown that exposure of treated WWTP effluent or TCS-contaminated water can transform TCS into various higher-chlorinated TCS derivatives, dioxins and benzofurans, including dichlorodibenzo-*p*-dioxins (DCDDs) and 2,4-DCP, when aqueous TCS is irradiated with sunlight/UV light, even at relatively low irradiation intensities [103,104,105,106,107]. Depending on the conditions, 3 to 12% of TCS was converted to DCDDs and 2,4-DCP, respectively, with some TCS being coupled to humic matter [108]. Furthermore, the production of chloroform and other disinfection byproducts can be significantly enhanced at higher pH values or when UV is combined with chlorine treatments and/or seawater [100,103,109,110].

### 2.3. Fate of Triclosan and Its Transformation/Degradation Products and Metabolites during Wastewater Treatment

Wastewater undergoes a variety of different treatments and conditions (i.e., aerobic, anaerobic, anoxic, high/low pH and/or reduction/oxidation potentials, etc.) as it is processed in a WWTP. For example, wastewater undergoes a variety of oxidation-reduction potentials in BNR processes, during which different microorganisms are active and a wide range of metabolic processes are occurring. A portion of antimicrobials can be degraded and/or transformed into other compounds during treatment.

Various studies have reported TCS concentrations of 0.3 to 12.5 µg/L in domestic wastewater [41,42,70,111,112,113,114]. Trace amounts of MeTCS in untreated wastewater have also been reported, with values between 0.015 to 1 µg/L [41,42,70,112,113]. TCS is largely removed from the aqueous phase of wastewater during treatment, as studies that analyzed WWTP effluents have demonstrated near-complete TCS removals ranging between 85 and 98% [39,40,41,42,56,70,115,116,117,118]. Despite high levels of removal from the aqueous phase of wastewater, only a fraction of this is due to TCS being fully or partially mineralized [31]. Numerous studies have shown that 66 to 80% of TCS is associated with particulate or solid matter in wastewater [8,39,42,116]. Soluble TCS tends to partition (or sorb) to particulate matter/sludge and can accumulate to concentrations several magnitudes higher during wastewater treatment [42,119]. This is likely due to the very high surface areas of wastewater sludge available for sorption (ranging from 0.8 to 1.7 m^2^ per gram of material) and the highly hydrophobic and lipophilic nature of TCS [31,75]. Significant TCS removals have been observed during primary settling through sorption and settling of solids, with up to 75% TCS removals being observed from the aqueous phase, although little to no biotransformation during primary treatment was observed [42].

Triclosan has been shown to degrade under aerobic conditions, and a wide range of biotransformation values during secondary (biological) treatment have been observed. Guerra et al. [111] found that TCS removal in WWTPs was strongly correlated with total Kjeldahl nitrogen removals. Some laboratory studies have shown high (75 to 99%) TCS degradation during simulated activated sludge processes [85,120], although full-scale studies typically find significantly lower removals. For example, Lozano et al. [42] observed 10% and 23% TCS removal during conventional activated sludge and subsequent nitrification/denitrification processes, respectively, while Heidler and Halden [39] observed just under 50% removal. This is likely due to a significant portion of TCS not being exposed to aerobic conditions due to removal during primary treatment as well as incomplete mixing or shortcutting during secondary treatment. Chen et al. [85] observed that approximately 1% of TCS in a simulated activated sludge process was transformed into MeTCS during aerobic conditions while no TCS removal or MeTCS generation occurred under simulated anoxic or anaerobic conditions. A simulated batch activated sludge process produced various monoaromatic compounds, including 4-CC and 2,4-DCP and several isomers of mono- and di-hydoxy-TCS, and approximately 7% of the initial TCS was transformed into TCS-SO_4_, which accumulated and persisted until the end of the experiment [57]. TCS removals in secondary clarifiers are primarily due to the settling of TCS-bearing biomass, and little change was reported in aqueous-phase TCS or MeTCS concentrations [39,42]. Although several studies have found that TCS can be transformed during chlorination or through UV disinfection processes, Lozano et al. [42] did not find any significant changes in dissolved TCS, and MeTCS remained constant during filtration and disinfection processes. Typical literature values in treated effluent range from 0.07 to 6.20 µg/L for TCS and from 0.015 to 0.02 µg/L for MeTCS [41,42,70,112].

Relatively high concentrations of TCS and MeTCS have been reported in primary and secondary sludge as they are the primary sinks for TCS and TCS-related compounds. A survey of six Canadian WWTPs published in 2015 found primary sludge TCS concentrations of 6 to 14 µg/g dw, while a 2009 study of a large U.S. facility reported average primary sludge TCS concentrations of 20.3 ± 0.9 µg/g dw and 0.09 ± 0.002 µg/g dw of MeTCS [42,121]. These studies reported somewhat lower concentrations in thickened waste activated sludge, with TCS values of 0.43 to 14.14 µg/g dw and 0.25 ± 0.04 µg/g dw MeTCS in the U.S. study (ibid). Mixed primary and secondary sludge TCS concentrations reported in the literature vary widely, with average values between 4.0 and 12.9 µg/g dw in several Canadian WWTPs [43,80,122,123,124], which were comparable to values found in the U.S. and elsewhere [125]. Other than MeTCS, few studies have found significant levels of other TCS metabolites in undigested sludge from full-scale facilities, with the exception of Abbott and Eskicioglu [80] who reported 0.1 to 0.2 µg/g of TCS-SO_4_ in undigested mixed sludge from a small- to medium-scale BNR facility. Table 2 presents the occurrences of TCS and its transformation products/metabolites in sludge and in various stabilization processes.

Many emerging contaminants are somewhat resistant to degradation and typically degrade at much lower rates than that of organic matter during sludge stabilization processes. Due to this, effluent from many stabilization processes, such as anaerobic digestion, often contain far higher antimicrobial concentrations (in terms of µg/g dw) and appear to be concentrated as sludge volumes that are typically reduced by 50% or more. Depending on the method of calculation, many studies have found negative TCS removals, and some refer to this as “accumulation” during digestion. For example, McAvoy et al. [41] found that anaerobic digestion was ineffective in degrading TCS in primary sludge with significant (108%) TCS accumulations in anaerobic digester digestate (15.6 µg/g dw). This study also observed the generation of small amounts of MeTCS (0.13 µg/g dw) and some (0.09 to 0.11 µg/g dw) higher-chlorinated TCS derivatives, including 2,3-dichloro-6-(2,4-dichlorophenoxy) phenol and 2,3,4-trichloro-6-(2,4-dichlorophenoxy) phenol.

Little TCS removal was also observed in several Canadian WWTPs employing anaerobic digestion. Guerra et al. [111] found low levels of TCS removals during digestion with average TCS concentrations of 10.7 µg/g dw in anaerobically digested biosolids from a conventional activated sludge facility. A bench-scale study using mixed primary and secondary BNR sludge (33:67% by mass) by Kor-Bicakci [122,124] observed significant increases in TCS concentrations (greater than two-fold in terms of µg/g dw) between anaerobic digester influent and effluent in both mesophilic (35 °C) and thermophilic digesters (55 °C). Digesters were operated at SRTs of 6, 12 and 20 days (corresponding to an organic loading rate (OLR) range from 1.46 to 5.25 g volatile solids (VS)/L/day) and showed less of an increase in TCS concentration (in terms of µg/g dw) in thermophilic versus mesophilic digesters. This agrees with results from full-scale mesophilic anaerobic digesters (~35 °C, ~21-day SRT) in Saskatchewan, Canada, which also utilized mixed primary and secondary BNR sludge [43]. Abbott and Eskicioglu [80] observed 3.0 to 37.3% TCS reductions in mesophilic anaerobic digesters utilizing mixed primary and secondary BNR sludge operated at SRTs ranging between 8 and 20 days (OLR of 1.9 to 4.76 kg VS/m^3^/day), with clear correlations between increasing SRT and TCS removals (R^2^ = 0.89). This study also reported anaerobic digester effluent TCS-SO_4_ levels of 0.09 to 0.19 ng/g dw and significant removals during anaerobic digestion of up to a 72% removal during the longest 20-day SRT and trace amounts of the TCS metabolite 2,4-DCP.

Aerobic sludge digestion has been shown to be more far effective than anaerobic digestion in degrading antimicrobials. The same study by McAvoy et al. [41] found that aerobic digestion of primary sludge was able to decrease TCS sludge concentrations by up to 88% and did not generate detectible levels of any higher-chlorinated TCS derivatives. Guerra et al. [111] also observed significant TCS removals during aerobic digestion and produced the lowest TCS biosolid levels (median value of 0.56 µg/g dw). Abbott and Eskicioglu [80] reported low (1.183 µg/g dw) TCS concentrations in effluent from cycling aerobic/anoxic sludge digesters and up to 80.3% TCS removal during the longest 20-day SRT (OLR of 4.76 kg VS/m^3^/day). They also reported a clear correlation between increasing TCS removals and digester SRT (R^2^ = 0.88) and near-complete (86 to 95%) removals of TCS-SO_4_, even at the shortest (5-day) SRT (OLR = 7.61 kg VS/m^3^/day) and did not detect any other TCS metabolites.

Some studies have also shown that various types of sequential digestion can also be effective in degrading TCS and can improve TCS removals versus comparable single-stage digesters. Samaras et al. [133] found that a temperature-phased anaerobic digester with a 12-day SRT thermophilic (55 °C) first stage combined with an 8-day mesophilic (37 °C) second stage was able to remove up to 82% of TCS across the system. Combinations of anaerobic and cycling aerobic/anoxic digesters have also been shown to be highly effective in degrading TCS. Tomei et al. [134] reported up to a 38.9% TCS removal across a 15-day SRT mesophilic (37 °C) anaerobic digester combined with a 12-day SRT mesophilic (37 °C) aerobic/anoxic digester. Abbott and Eskicioglu [80] found that up to 64.5% of TCS and up to 96.4% of TCS-SO_4_ were removed across mesophilic anaerobic digesters when combined with mesophilic aerobic/anoxic digesters operated at total SRTs of 20 days. In this study, they did not detect any other TCS metabolites.

### 2.4. Fate and Impacts of Triclosan and Triclosan-Related Compounds in the Environment

Triclosan is commonly reported as one of the top trace contaminants in the environment [5,31,125]. It is found widely in surface waters, in sediments which receive treated WWTP effluent and in soils receiving biosolids (ibid). TCS contains multiple halogen atoms and is highly xenobiotic; thus, many microorganisms lack the necessary metabolic pathways and enzymes to degrade it. This results in it being highly persistent and bioaccumulative in the environment [31]. It had long been assumed that antimicrobials released to the environment were not present in sufficient concentrations to have significant health and/or environmental effects. However, it is becoming increasingly clear that even trace levels of TCS can have significant impacts on aquatic ecosystems and may alter the function of soils [135,136].

Triclosan can alter the function of aquatic environments and can have toxic effects on a variety of species, suggesting that aquatic ecosystems may be significantly altered by TCS contamination. For example, saltwater shrimp (*Palaemon varians*) have been shown to avoid TCS-contaminated areas at concentrations as low as 10 µg/L using both linear and heterogeneous multi-habitat assay systems [137]. TCS is also directly toxic to many aquatic organisms and has both acute and chronic effects, with the neutral form showing the greatest toxicity [103,138,139]. TCS has been shown to have sublethal effects on a variety of aquatic species at its lowest observed effect concentration of 71.3 µg/L for *Oncorhynchus mykiss* (rainbow trout), and a 48-h median effective concentration of 390 µg/L for *Daphnia magna* [138]. TCS also has acute toxic effects at the median lethal concentration (LC50) of TCS 0.48, 0.47 and 1.67 mg/L for fish, daphnia and green algae, respectively according to an ecological structure activity relationships model [139]. Direct laboratory testing has shown a wide range of 96-h LC50 values of 0.497 mg/L for *Litopenaeus vannamei* (shrimp) to 1.403 mg/L for *Oreochromis niloticus* (tilapia), 260 mg/L for *Pimephales promelas* (minnow) and 370 mg/L for *Lepomis macrochirus* (bluegill) [136,138]. Threshold values for chronic toxicity of TCS in fish and crustacea were found to be as low as 34–290 and 6–182 µg/L, respectively [140]. The 25% inhibition concentrations (IC25) reported were 290 µg/L for fish (*Oryzias latipes*) and 170 µg/L for Crustacea (*Ceriodaphnia* sp.) [141].

Orvos et al. [138] observed significant TCS accumulation within biota, with *Danio rerio* (zebrafish) showing a 5-week bioaccumulation factor of 4157 at aquatic system TCS concentrations of 3 µg/L. Adolfsson-Erici et al. [142] also observed significant TCS accumulation in the bile of wild-living fish downstream of WWTPs, with bile TCS concentrations of up 4.4 mg/kg of fresh weight for *Rutilus rutilu* (common roach fish) and concentrations of up to 47 mg/kg of fresh weight in caged *Oncorhynchus mykiss* immediately (0 km) downstream of a Swedish WWTP.

Triclosan is also persistent in soils. Modeling of antimicrobials in aerobic soils suggests that TCS has a half-life of 18 days, while no appreciable degradation was found even after 70 days in anaerobic soils [143]. Aerobic laboratory studies of different soil types indicated TCS has a half-life of 20 to over 100 days [132,144], while MeTCS has been shown to be significantly more persistent with a half-life of 443 days [132]. TCS at levels below 10 mg/kg has been shown to disturb the nitrogen cycle in soils using a substrate-induced respiration assay, while TCS levels of 0.1 to 50 mg/kg were found to inhibit soil respiration and phosphatase activity using respiration and phosphatase activity tests [145,146].

Although many studies have been unable to find direct toxic effects of TCS at levels commonly found within humans, there are several other concerns [31]. For example, TCS has been shown to reduce the effectiveness of several important human antibiotics by up to 100-fold in vivo [65] and it has been shown to significantly alter human gut and skin microbiomes [147,148]. TCS has structural similarities to several thyroid hormones and has been shown to have dose-dependent effects on thyroid hormone production in animal studies [149]. Kim et al. [150] and Park et al. [151] both showed that perinatal TCS exposure caused porcine mitochondrial disruption and oxidative stress. Tran et al. [152] demonstrated that perinatal TCS exposure induced neurodevelopment disorders in mice, leading to significant social, cognitive and learning defects in offspring mice.

In vitro, TCS has been also shown to be a potent inhibitor of enzymes which are responsible for the detoxification of multiple industrial chemicals by blocking the genetic transcription of several key human liver enzymes. These enzymes are responsible for the “biotransformation of steroids, pharmaceuticals and other xenobiotics” [38,72,153]. TCS has also been shown to cause significant oxidative stress to the human liver and there are concerns about its hepatocarcinogenic potential [154]. In vivo, TCS has been shown to significantly increase the rate of hepatocellular carcinoma in mice, as it can promote the formation, size and propagation of liver tumors [69]. There is also concern regarding many common TCS contaminants and degradation byproducts, many of which are acutely toxic and/or carcinogenic, even at extremely low levels [31].

In humans, TCS is primarily excreted in feces and in urine [59,155]. Urinary excretion of TCS conjugates is the predominant means of elimination in people, the bulk of which occurs within the first 24-h of exposure [38,59,156]. Despite relatively quick removal, significant concentrations of TCS have been found in humans. A North American study conducted between 2005 and 2008 found TCS in the urine of 80% of randomly selected people with TCS concentrations of 2.3 to 3620 ng/mL, with a geometric mean of 23.1 ng/mL (*n* = 3041) [148]. A more recent (2009 to 2013) and larger (*n* = 8195) Canadian study found TCS urine concentrations with a geometric mean of 16 ng/mL of TCS, with 71% of children (3 to 19 years old) and 69% of adults (20 to 79 years old) having detectable concentrations [157]. TCS is also commonly found in human tissues and bodily fluids, including breast milk [38,56,142,158], amniotic fluid [59], donated blood plasma [155], semen [159] and urine and feces [156,157,159]. Chronic and sub-lethal TCS levels have been shown to promote antibiotic resistance in human pathogenic bacteria. TCS induced high levels of antibiotic tolerance in vitro at environmentally relevant concentrations and was shown to reduce the effectiveness of antibiotics by up to 100-fold in vivo against *E. coli* and MRSA in animal studies [65]. Other studies have shown significant relationships between TCS concentrations and the relative abundance of antibiotic-resistant genes in dust inside homes [160]. Studies have also shown increases in antibiotic-resistant genes and changes in community structure favoring human pathogenic bacteria inside anaerobic digesters [161].

While a large number of studies have been conducted which have explored potential human health risks and ecological impacts of TCS, far less is known about the potential impacts of its conjugates, transformation and degradation products [162,163]. This is partially due to the highly complex wastewater, sludge and environmental sample matrixes involved, and the challenging and expensive extraction and analysis methods required to detect and quantify these compounds [164]. The literature, to date, has shown that several of these compounds are persistent in the environment [139], while many, including 2,4-DCP, trichlorophenols and chloroform, are listed on the U.S. Environmental Protecion Agency (U.S. EPA) Priority Pollutant List and are regulated by the U.S. Clean Water Act [108]. MeTCS has been shown to contribute to the toxicity of treated wastewater effluent and is directly toxic to (or can impact) the embryonic development of a variety of organisms, including *Vibrio fischeri*, zebrafish (*Danio rerio),* sea urchins (*Paracentrotus lividus)* and European abalone (*Haliotis tuberculata*) [165,166,167]. Furthermore, several partial TCS mineralization products, including phenol, chlorohydroquinone and hydroquinone, are known to be toxic to humans and/or many aquatic species. Chloroform, trichlorophenols and catechols are also highly toxic to humans and animals and are suspected or known carcinogens [168,169], while DCDDs are environmental contaminants which are well known to be particularly persistent, toxic and highly carcinogenic [139]. Although the direct impacts of TCS are becoming better understood, further work is urgently needed to better understand the potential impacts of its conjugates and degradation products and their fates in the environment.

## 3. Legacy Antimicrobial Triclocarban

### 3.1. Background

**Figure 5:**
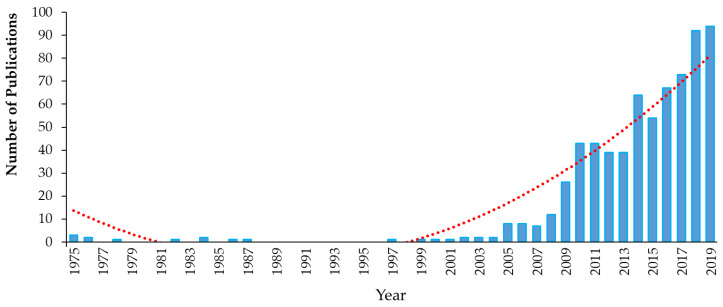
Number of scientific documents on the topic of “triclocarban” published for the time period from 1975 to 2019 (data generated from reference [74]).

Triclocarban (3-(4-chlorophenyl)-1-(3,4-dichlorphenyl)urea; CAS #101-20-2) is a broad-spectrum antibacterial and antifungal agent that is mostly active against Gram-positive bacteria, such as MRSA, vancomycin-resistant enterococci and *Streptococcus* [31,170,171]. Since 1957, TCC has been commonly added to many consumer and personal care products as an active ingredient at levels of 0.5–5% (by weight) for preventing spoilage and microbial infections through the disturbance of microbes’ fatty acid synthesis and cell membrane formation [119,172,173]. TCC has been present in over 2000 different products, such as detergents, bar soaps, body washes, toothpaste, cleansing lotions, deodorants and many other cosmetics [31]. TCC has been categorized as a high-production-volume chemical (annual production or importation volume between 250 and 500 metric tonnes) by the U.S. EPA [174]. In 1999–2000, TCC was found in 29% of bar soaps, formulated to concentrations of about 2% (by weight) in the U.S. market [31]. More than 650 scientific papers have been published on the topic of “triclocarban” between 1975 and 2019 (Figure 5).

The antimicrobial TCC is a trichlorinated binuclear aromatic compound which has toxic, persistent and bioaccumulative characteristics [31,175]. Regarding its physico-chemical properties (Table 3), TCC has a high Log K_ow_ value and a dissociation constant (pKa), while it has low water-solubility [144,176]. TCC has emerged as one of the most frequently detected priority contaminants in aqueous (e.g., wastewater, surface water and drinking water) and solid matrices (e.g., sludge, biosolids, soils and sediments) due to its direct discharge and/or incomplete removal in WWTPs [5,18,20,29,176,177,178].

### 3.2. Transformation/Degradation Products and Metabolites of Triclocarban

Partial elimination of TCC in WWTPs or in the environment has led to the formation of several transformation/degradation products and metabolites as a result of various abiotic and biotic mechanisms, such as microbial metabolism, hydrolysis, oxidation and photolysis. Among TCC’s potential degradative intermediates and/or metabolites detected in aquatic and terrestrial ecosystems, the most common compounds are chlorocarbanilide congeners (dichlorocarbanilide (DCC), monochlorocarbanilide (MCC) and carbanilide (NCC)), chloroanilines (3,4-dichloroaniline (3,4-DCA) and 4-chloroaniline (4-CA)), 4-CC, catechol and aniline [170,173,179,180,181,182,183,184,185]. Microbial degradation has been considered as one of the main mechanisms for TCC transformation and elimination, especially in wastewater treatment. However, so far, only a few studies have explored the transformation/degradation pathway of TCC by isolated microorganisms from mixed microbial consortia in activated sludge. The degradation pathway of TCC by a pure bacterium *Sphingomonas* sp. strain YL-JM2C, which was isolated from activated sludge of a WWTP in Xiamen (China), was studied by Mulla et al. [170]. This microorganism transformed TCC (up to 35% of the initial concentration) into chloroanilines, and further into 4-CC. This bacterium was also able to degrade TCC’s intermediates, including 3,4-DCA and 4-CA [170]. Another study by Miller et al. [180] showed that a wastewater bacterium, belonging to the family Alcaligenaceae, was involved in the degradation of TCC and its non-chlorinated congener, NCC. This wastewater bacterium was found to be capable of using these compounds as its sole carbon and energy source. Yun et al. [186] also investigated the transformation of TCC by *Ochrobactrum* sp. TCC-1 in wastewater sludge under anoxic nitrate respiration conditions. According to their results, enhanced biotransformation of TCC to chloroanilines could be supported by nitrate under anoxic conditions. TCC degradation and its metabolites’ production during TCC removal from wastewater were also evaluated using entrapped *Pseudomonas fluorescens* strain MC46 in the study by Taweetanawanit et al. [185]. In their study, while TCC concentration decreased dramatically in the first 6 h of 8-h experiment, intermediate products (i.e., 3,4-DCA and 4-CA, and aniline) were produced at low levels and further degraded. On the other hand, Sipahutar et al. [183] explored the efficiency of a soil bacterium, *Pseudomonas fluorescens* MC46, on TCC removal in contaminated soil. TCC was efficiently removed (up to 74–76% of the initial concentration) from soil by transforming into 3,4-DCA and aniline. According to study by Sipahutar and Vangnai [184], a soil-inhabiting bacterium *Ochrobactrum* sp. MC22 was found to be capable of degrading TCC and its chloroaniline metabolites under both aerobic and anaerobic conditions. In the study by Liang et al. [187], TCC and its dechlorinated congeners (DCC, MCC) were effectively removed from contaminated soil by the bioaugmentation of TCC degrader *Ochrobactrum* sp. TCC-2 and/or chloroanilines degrader *Diaphorobacter* sp. LD72. Their hydrolysis products (chloroanilines) were also further degraded by these functional degraders. Furthermore, functional characterization of a novel amidase involved in biotransformation of triclocarban in *Ochrobactrum* sp. TCC-2, isolated from a river sediment sample, was studied by Yun et al. [182]. A novel amidase gene was found to be responsible for the hydrolysis of TCC and its dehalogenated congeners DCC and NCC to more biodegradable chloroaniline or aniline products, under anaerobic denitrifying conditions.

**Figure 6:**
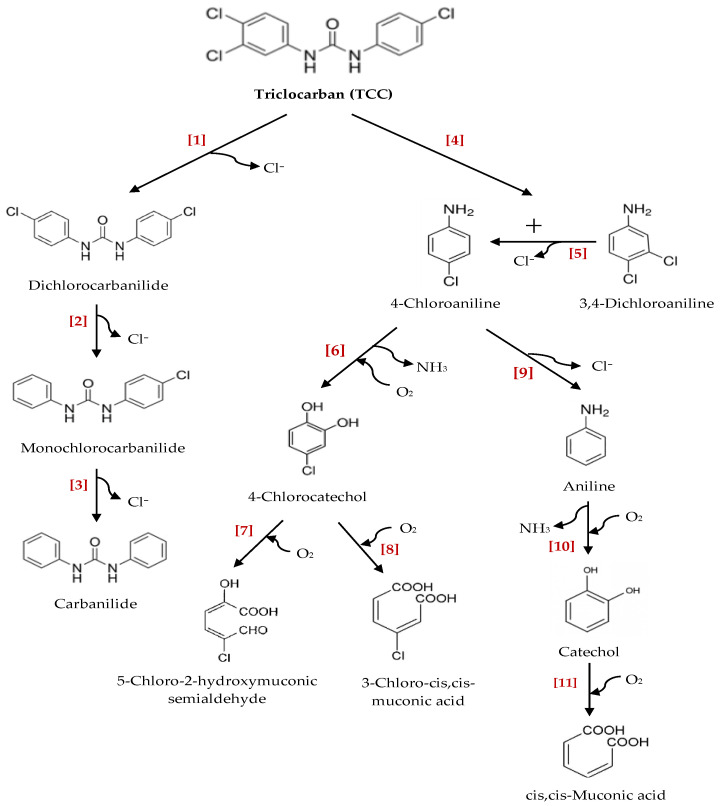
Proposed pathways for the potential transformation/degradation of triclocarban by microorganisms. The reactions are denoted as follows: [1–3] reductive dechlorination; [4] hydrolysis; [5] dechlorination; [6] deamination and hydroxylation; [7] *meta*-cleavage; [8] *ortho*-cleavage; [9] dechlorination; [10] deamination and hydroxylation; [11] *ortho*-cleavage (adapted from references [170,173,179,180]).

Proposed pathways for the potential transformation/degradation of TCC are shown in Figure 6, taking the above-mentioned studies into account. As a summary, the hydrolysis is found to be the first step of the biodegradation pathway during the transformation of TCC into chloroanilines, namely 3,4-DCA and/or 4-CA [170,180,184,185,188,189]. Subsequently, 3,4-DCA is transformed into 4-CA via dechlorination [170,180]. In the next step, 4-CA is further degraded to 4-CC by deamination and hydroxylation [170,180]. Intermediate 4-CC may then be converted into 3-chloro-cis,cis-muconic acid or 5-chloro-2-hydroxymuconic acid semialdehyde through *ortho*- or *meta*-cleavage pathways, respectively [180,190,191]. According to the study conducted by Hongsawat and Vangnai [179], a bacterium *Acinetobacter baylyi strain* GFJ2 isolated from soil also has another degradation pathway that produces aniline from 4-CA. Aniline is then converted into catechol, which may further be degraded via an *ortho*-cleavage route, producing cis,cis-muconic acid (Figure 6).

On the other hand, reductive dechlorination is also known to be a ubiquitous and essential dissimilation route that takes place during the transformation of TCC and its higher chlorinated congeners (such as 3,3’,4,4’-tetrachlorocarbanilide (4-Cl-TCC)) into their lesser chlorinated congeners under anaerobic and anoxic conditions by the activity of microorganisms [56,119,192,193,194]. Through sequential detoxification reactions, TCC can be converted to its core carbanilide structure as follows: Tri-chlorinated TCC is first transformed into DCC, then into MCC and, finally, into non-chlorinated NCC (Figure 6) [56,188,192]. However, the transformation of DCC into MCC is generally considered rate-limiting during dechlorination process [162,195].

Regarding the metabolism of TCC by higher animals, TCC was significantly metabolized in rats, monkeys and humans and then excreted in bile, feces and/or urine. Previous studies identified five metabolites of TCC (including 2′-hydroxy-TCC, 3′-hydroxy-TCC, 6-hydroxy-TCC, 2′,6-dihydroxy-TCC and 3′,6-dihydroxy-TCC) after oral, intravenous or dermal administration of ^14^C-labelled TCC to higher animals [196,197,198]. Schebb et al. [34] measured human urine concentrations 0–72 h after showering with a commercial bar soap containing 0.6% TCC in order to evaluate human exposure to TCC. Their results showed that a large amount of TCC was excreted as *N*-glucuronides via urine.

### 3.3. Fate of Triclocarban and Its Transformation/Degradation Products and Metabolites during Wastewater Treatment

As TCC is relatively non-volatile (Table 3), photodegradation and/or volatilization mechanisms are not expected to be the main removal pathway for TCC during wastewater and sludge treatment. Consequently, the removal of TCC from the aqueous phase may be either an outcome of biological processes or sorption to the sludge phase. To date, several studies in the literature have investigated the occurrence, fate and distribution of TCC in both aqueous and solid waste streams in WWTPs. According to their findings, TCC has shown a high tendency to partition onto sludge during wastewater treatment because of its lipophilic and hydrophobic properties [9,199,200]. Although a significant portion (up to 99%) of TCC has been removed from the aqueous phase of treated wastewater, TCC has been observed to accumulate in untreated (e.g., primary, secondary and mixed) and/or treated (e.g., digested, dewatered and composted) sludges at concentration levels of ng/g to μg/g dw [32,42,119,201,202,203]. Heidler et al. [119] reported average TCC concentrations of 6100 ± 2000 ng/L and 170 ± 30 ng/L in the influent and effluent, respectively, in a conventional U.S. WWTP. According to their mass balance study, approximately 21 ± 30% of the initial mass of TCC was transformed during wastewater treatment and about 3 ± 1% of TCC was passed through the plant into effluent-receiving surface water, whereas 76 ± 30% of TCC accumulated in municipal sludge. Even though the removal efficiencies of TCC from the aqueous phase of four U.S. WWTPs were between 80 and 99%, it accumulated in the sludge at up to 1425 ng/g dw [203]. Additionally, Lozano et al. [42] revealed that the highest TCC removal rate from the aqueous phase was achieved during primary treatment of a large U.S. WWTP. In the following stages, TCC levels did not change during the secondary treatment, but approximately an 18% TCC decrease was observed during the nitrification and denitrification processes. According to their mass balance study, a large fraction of TCC (>79%) was transferred to the biosolids. Among the 48 target PPCPs studied by Sun et al. [199], TCC was detected in all of the influent, effluent and sludge samples (*n* = 12) collected from three WWTPs located in different areas in China over one year. Average TCC concentrations in the influent and effluent ranged from 4.7 to 109 ng/L, whereas TCC levels in sludge were in the range of 1130–2180 ng/g dw with an average of 1700 ng/g dw. The occurrence of TCC in aqueous and solid waste streams of seven Chinese WWTPs was investigated in another study by Wang et al. [9]. Although dissolved TCC had relatively low concentrations (between 4.78 and 500 ng/L, with detection frequencies of >95.9%) in WWTP influent and effluent samples, adsorbed TCC was detected in all suspended solids and sludge samples (*n* = 49), with median concentrations of 968 and 1090 ng/g dw, respectively. On the other hand, among the 19 biocides monitored in eight municipal WWTPs in Thailand, TCC was determined as the predominant compound in the influent, effluent and excess sludge, with average concentrations of 838 ng/L, 95.6 ng/L and 4010 ng/g dw, respectively [29]. These studies clearly indicate that the sorption of TCC to sludge was found to be the main mechanism in its removal during wastewater treatment.

TCC has been identified as one of the top trace contaminants among wide-ranging PPCPs in sludge and biosolids assayed in several countries, such as the U.S. [18,201,204], Canada [205], China [32,206], Japan [117], Korea [207] and Thailand [29]. TCC was found to be one of the most abundant analytes (48% of total PPCP mass found) among the 72 PPCPs detected in biosolids samples obtained from 94 U.S. WWTPs in 32 states and the District of Columbia, and its mean concentration was 36,060 ± 8049 ng/g dw [18]. The occurrence of TCC within 72 PPCPs assayed in sludge samples taken from 74 facilities was also investigated by the U.S. EPA in its 2009 “Targeted National Sewage Sludge Survey”. TCC was detected in all of the sludge samples at concentrations ranging from 187 to 441,000 ng/g dw [125]. Moreover, the levels of TCC in lime-stabilized biosolids samples, which were taken from a WWTP in the Mid-Atlantic region of the U.S. at around every 2 months over a 7-year period, were between 8850 and 22,900 ng/g dw, with an average concentration of 14,300 ± 3710 ng/g dw (*n* = 31) [33]. TCC was also identified as a priority compound among the top 10 contaminants from the 231 emerging chemicals analyzed in the U.S. biosolids regarding its high occurrence and high bioaccumulation potential [5]. A study conducted by Guerra et al. [205] reported that TCC was present in every biosolid sample (*n* = 24) collected from six WWTPs in Canada and was one of the most abundant compounds, at concentrations from 1200 to 8900 ng/g dw, among the 62 PPCPs. Another study by Chu and Metcalfe [208] reported that TCC concentrations in samples collected from three Canadian WWTPs were between 2170 and 4820 ng/g dw in activated sludge and between 3050 and 5970 ng/g dw in biosolids. In the study by Liu et al. [32], TCC was also found to be the predominant compound within 18 biocides analyzed in 10 WWTPs in South China and its concentration levels in excess sludge were between 1140 and 2950 ng/g dw. According to the first nationwide study in Korea performed by Subedi et al. [207], TCC among 10 PPCPs was found in 100% of digested sludge samples taken from 40 representative WWTPs that receive domestic, industrial or mixed (both domestic and industrial) wastewaters. Their results showed that the median concentrations of TCC in digested sludge collected from domestic (2860 ng/g dw) and mixed (2380 ng/g dw) WWTPs were 160–200-times greater than that in digested sludge collected from industrial WWTPs (14.2 ng/g dw). Lehutso et al. [202] also reported TCC concentrations in raw sludge, treated sludge and biosolids ranging between 3.58 and 11.8, 1.95 and 8.31 and 2.59 and 11.9 ng/g dw, respectively, in six WWTPs of South Africa over a period of two years.

As the anaerobic digestion process is a commonly used sludge stabilization option in municipal WWTPs because of its capability by generating bioenergy and biosolids [209], several studies have focused on the occurrence and fate of persistent TCC during conventional anaerobic sludge digestion. In the study by Heidler et al. [119], a mesophilic (35–37 °C) digester operated at an SRT of 19 days did not improve TCC transformation and resulted in an accumulation of TCC in dewatered, digested sludge up to concentrations of 51,000 ± 15,000 ng/g dw. Another study indicated the accumulation and persistence of TCC in anaerobically digested biosolids [201]. Furthermore, Yang et al. [210] observed no TCC degradation during anaerobic digestion operated under mesophilic (35.0 ± 0.5 °C) conditions at SRTs of 15, 20 and 30 days and TCC remained predominantly in the solid phase of digested sludge in their study. Kor-Bicakci et al. [44] also reported that anaerobic digesters fed with mixed sludge at different SRTs of 20, 12 and 6 days, showed limited TCC removals (from 11 to 32%) under mesophilic (35 ± 1 °C) and thermophilic (55 ± 1 °C) conditions. Narumiya et al. [117] showed that the level of TCC was reduced moderately during anaerobic digestion in a Japanese WWTP and was found at one of the highest concentrations (from 1540 to 3140 ng/g dw) in digested sludge among the 47 PPCPs examined. Consequently, it can be concluded that the transformation/degradation of TCC is very limited during the conventional anaerobic digestion. For this reason, recently, a few studies have focused on solutions (such as using sludge pretreatment technologies prior to digestion) that can boost the removal of TCC and its potential degradation products from digested biosolids. Yang et al. [211] reported that anaerobic sludge digestion using thermal pretreatment at a temperature of 150 °C for 30 min (with a pressure of 500 kPa) enhanced the biodegradation of TCC by around 10%. The impact of the Cambi Thermal Hydrolysis Process™ in conjunction with mesophilic anaerobic digestion (at an SRT of 22 days) on the levels of TCC and its transformation products in biosolids was investigated by Armstrong et al. [127]. The implementation of thermal hydrolysis, applied at temperatures of 150–180 °C for 30 min, to the full-scale WWTP allowed for a significant decrease in the levels of TCC (from 5734 ± 2182 to 630 ± 974 ng/g dw) from biosolids. In another study by Kor-Bicakci et al. [44], advanced anaerobic digestion coupled with microwave pretreatment was found to be highly efficient in increasing TCC elimination from digested biosolids. TCC was eliminated significantly (from 43 to 78%) in the advanced digesters fed with microwave-pretreated sludge at 160 °C, while advanced digesters fed with microwave-pretreated sludge at 80 °C displayed moderately efficient TCC removal (from 17 to 47%) from digested biosolids. According to their results, within the OLR range of 1.46 to 5.25 g VS/L/day, thermophilic (55 ± 1 °C) digesters had better performances in the reduction in TCC in biosolids compared to mesophilic (35 ± 1 °C) digesters. Furthermore, Yu et al. [186] indicated that the biodegradation rate of TCC was 83.1% after composting of sludge for 16 days with forced ventilation, whereas this rate was only 18.2% after sludge stacking for 16 days.

Although most studies have investigated the occurrence and fate of TCC in sludge and biosolids, only a few have reported its behavior coupled with its potential transformation products and metabolites [44,56,127,201,206]. This can be explained by the lack of accurate and cost-effective analytical methods for the quantitative analysis of complex sludge matrix and the challenges encountered during their analysis [164,212]. Table 4 shows studies that reported the occurrences of both TCC and its transformation products/metabolites in sludge and biosolids. Sapkota et al. [213] revealed that both DCC and 4-Cl-TCC, which were previously unrecognized pollutants, were detected consistently in primary sludge samples from various WWTPs located in five different U.S. states beside TCC. In their study, high Log K_ow_ values (4.3 and 5.6, calculated using the K_ow_ WIN^TM^ model) and low water solubilities (3.7 and 0.11 mg/L, calculated using the Solaris V4.67 model) were reported for DCC and 4-Cl-TCC, respectively. Based on the quantitative structure–activity relationship analyses, DCC was found to be less particle-active and 34-times more water-soluble compared to 4-Cl-TCC [213]. In another study conducted by Heidler and Halden [201], 4-Cl-TCC and DCC were detected for the first time in anaerobically and aerobically digested sludge collected from 25 full-scale WWTPs in 18 U.S. states. In their study, TCC was found at comparatively higher levels compared to its transformation products, detected in digested sludge. Moreover, the nationwide survey by Pycke et al. [56] was the first to report TCC transformation through dechlorination occurring in the sewage collection system and within a WWTP. DCC and MCC were detected in raw and digested sludge samples obtained from 14 different WWTPs from across the U.S. The effects of varying sludge treatment processes on the levels of TCC and its dechlorination products in biosolids were also investigated in the same study [60]. Anaerobic digestion processes caused significant accumulation of these compounds in digested sludge, whereas digested sludge heat drying processes enhanced the removal of TCC, DCC and MCC. Conversely, very limited removal efficiencies for TCC and 4-Cl-TCC were obtained during the digested sludge dewatering process, while no significant changes were observed for DCC and MCC in the same process. In the study by Chen et al. [206], TCC dechlorination during wastewater treatment was observed for the first time in China. Two human metabolites of TCC (2′-hydroxy-TCC and 3′-hydroxy-TCC) were also detected in over 90% of the 100 sewage sludge samples collected across 21 Chinese provinces/districts. In another study conducted by Kor-Bicakci et al. [44], TCC was able to be converted into its non-chlorinated congener (NCC) during conventional and advanced anaerobic digestion. Their research was the first to report the efficient and complete TCC dechlorination occurred in advanced thermophilic and mesophilic digesters fed with microwave-pretreated mixed sludge.

### 3.4. Fate and Impacts of Triclocarban and Triclocarban-Related Compounds in the Environment

There are significant concerns that TCC released to the environment through wastewater sludge may have potential adverse effects on human health and on ecosystems. Due to its high chlorine content and aromatic nature, TCC exhibits a tendency for environmental persistence [45,143,144,176,214,215]. The environmental half-life of TCC has been estimated at 60, 120 and 540 days in water, soil and sediment, respectively [176]. Laboratory experiments conducted by Ying et al. [143] showed that TCC was degraded in a loam soil with a half-life of 108 days under aerobic conditions, whereas it was highly resistant to biodegradation within 70 days of the experimental period under anaerobic conditions. The estimated half-life of TCC ranged from 87 to 231 days in soils with and without the addition of biosolids under aerobic conditions [144]. Another study also reported a soil TCC half-life of 287.5 ± 45.5 days in commercial farms that received a single biosolids application [215].

Several studies have explored potential endocrine-disrupting effects of TCC on animals (e.g., rats, frogs, zebrafish and sea urchins) [171,216,217,218,219] and humans [175,220,221]. TCC has also been shown to have detrimental impacts on aquatic organisms (e.g., algae, crustaceans and fish) [3,140,222] and on microorganisms [223,224]. In a study by Chalew and Halden [140], crustacea were identified as one of the most sensitive species to TCC, with acute and chronic toxicity threshold values between 0.0019 and 0.04 and 0.00006 and 0.0047 mg/L, respectively. In the same study, acute and chronic threshold levels in fish were observed in the range of 0.049–0.18 and 0.005 mg/L, respectively. According to the results of acute toxicity tests of TCC on eight Chinese aquatic species, *Daphnia magna* (planktonic crustacean) with a 48-h LC50 of 0.006896 mg/L was the most sensitive species to TCC, followed by *Rana chensinensis* (0.02384 mg/L, amphibian), *Gobiocypris rarus* (0,1103 mg/L, fish) and *Macrobrachium nipponense* (0.2616 mg/L, abenthic crustacean [222]). TCC also has acute toxic effects with a 48-h LC50 of 0.01, 0.0031 and 0.015 mg/L for *Daphnia magna*, *Ceriodaphnia dubia* and *Mysidopsis bahia* (invertebrates), respectively [174]. Moreover, the 96-h LC50 values of TCC to *Salmo gairdneri* and *Lepomis macrochirus* (fish) were 0.12 and 0.097 mg/L, respectively [174]. TCC has also been shown to bioaccumulate in terrestrial organisms (e.g., earthworms) inhabiting biosolid-amended soil ecosystems [14,225,226,227,228]. On the other hand, it has been found to accumulate in organisms (e.g., snails, freshwater worms, fish and algae) living in aquatic environments exposed to effluent from WWTPs [229,230,231,232,233]. Transfer of TCC from biosolid-amended soils and treated wastewater into aquatic and terrestrial ecosystems opens a pathway for potential bioaccumulation, bioconcentration and biomagnification of TCC throughout the food chain [30]. The study carried out by Sherburne et al. [226] demonstrated that biosolids-derived TCC had been found at different trophic levels within a terrestrial food web, including earthworms (primary consumer), deer mice (secondary consumer), European starlings (a secondary consumer of invertebrates) and American kestrels (a tertiary consumer of rodents, small birds and invertebrates). Furthermore, TCC has been found to bioaccumulate in plants (e.g., carrot, green pepper, tomato, cucumber, pumpkin, zucchini and switch grass) grown in soils amended with biosolids [28,48,52,53]. TCC was also found to be concentrated in root tissues and translocated into above-ground parts, such as stems, leaves and fruits [51,53,234].

Upon human exposure, TCC can be metabolized and excreted by the human body during its utilization in consumer and personal care products [34,35,235,236]. Schebb et al. [35] reported the detection of TCC in whole blood after exposing human subjects by a single shower with antibacterial soap. TCC has also been detected in many human matrices, including urine [34,56,172,237,238], serum [172], breast milk [33] and maternal urine and cord blood plasma [155,236,239], and in finger and toenails [237]. Ye et al. [172] measured the total and free concentrations of TCC and its phase-I metabolites (2’-hydroxy-TCC and 3′-hydroxy-TCC) in 50 urine and 16 serum samples collected anonymously from adult volunteers in the U.S. Total (free plus conjugated) TCC was detected in 5.4–28% of the urine samples together with its phase-I metabolites, whereas total TCC was detected in about 50% of the serum samples. Pycke et al. [155] also investigated the total concentrations of TCC and its human metabolites in maternal urine from 181 pregnant women in an urban population from Brooklyn, New York. Excreted TCC was detected in the urine of 86.7% of the study participants, while total 2′-OH-TC and 3′-OH-TCC were detected after conjugate hydrolysis in 27.1% and 16.6% of the urine samples, respectively.

Regarding transformation/degradation products of TCC, their potential ecological and human health risks are still rarely known [162,163,187]. TCC is known to be precursor of toxic and carcinogenic chloroanilines in the form of mono- and di-chloroaniline [31,192,201]. Specifically, 3,4-DCA has been recognized as an endocrine-disrupting chemical [240]. Furthermore, 4-CA has also been known to cause cancer [241]. Because of their recalcitrant properties, 4-CA and 3,4-DCA have been ubiquitously present in the environment and have been shown to accumulate in sludge, soil and sediments [179,242,243,244]. In a study by Sipahutar et al. [183], the toxicity of TCC and its degradative intermediates (3,4-DCA and aniline) were investigated using cytogenotoxicity assessments. According to an *Allium cepa* root-tip chromosomal aberration assay, root-tip cells exposed to TCC at various concentrations had more chromosomal abnormalities compared to root-tip cells exposed to TCC’s degradative intermediates. In addition to 4-chloroisocyanatobenzene and 4-chloronitrobenzene, 3,4-DCA and 4-CA were also identified as the main degradation products of TCC during photodegradation in aqueous systems under ultraviolet radiation [245] and simulated sunlight irradiation [246]. Concerning dechlorination products of TCC, DCC and 4-Cl-TCC are known as manufacturing byproducts in technical-grade TCC (at an estimated level of 0.2% by weight) [213]; thus, TCC-containing personal care products can be considered as another source of the release of these compounds into the environment [177]. The higher- and lower-chlorinated congeners of TCC have been reported to be present in sediments and surface waters impacted by WWTP effluent as well as in sediment-dwelling organisms, such as the freshwater oligochaete *Lumbriculus variegatus* (worm) [162,192,193,213,230,247]. A previous study conducted by Miller et al. [192] reported that the TCC dechlorination process was observed exclusively in strictly anaerobic environments (such as dated estuarine sediments) by strict anaerobic dechlorinating microorganisms. However, an alternate TCC dechlorination pathway was proposed in recent studies that took place in potentially partially oxygenated environments, such as freshwater sediments [162,195]. Furthermore, its carbanilide congeners have also been shown to act as endocrine disruptors [171]. Based on the few studies available in the literature, further research is required to better understand the potential impacts of these transformation/degradation products.

## 4. Emerging Alternative Antimicrobials

### 4.1. Introduction to Emerging Alternative Antimicrobials

**Figure 7:**
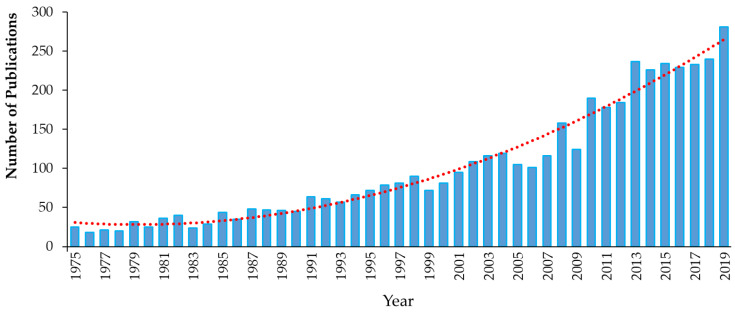
Number of scientific documents on the topic of “benzalkonium chloride” published for the time period from 1975 to 2019 (data generated from reference [74]).

Products containing emerging alternative antimicrobials are becoming increasingly common in the market place [129,248,249,250]. Most hand sanitizers in the present U.S. market contain emerging antimicrobials [251], while the recent novel coronavirus pandemic has also created an increasing demand for hand sanitizers and other disinfection products. This has created a huge market opportunity for manufacturers to continue to produce and market large amounts of antimicrobial-containing products [252]. Therefore, it is vitally important to investigate any potential environmental impacts and human health effects of these alternative antimicrobials, as the excessive use of them may lead to similar environmental and human health concerns as legacy antimicrobials. More than 4500 scientific papers have been published on the topic of “benzalkonium chloride” between 1975 and 2019 (Figure 7).

Over the last decade, benzyl-substituted quaternary ammonium chlorides (QACs) have become common in the marketplace as emerging alternative antimicrobials. The history of the use of QACs as a cleaning agent dates back to 1935, when Domagk reported the enhanced efficacy of the substituted long-chain alkyl groups [253]. In the beginning, quaternary ammonium salts were thought to be innocuous and green chemicals which possessed powerful germicidal and antimicrobial activities. Consequently, the extensive application of BACs in product formulations has resulted in the wide distribution of them in the environment, particularly in wastewater and sludge [254,255,256,257]. This section will focus on the environmental fate and distribution of BACs since they are one of the few classes of compounds which are currently not restricted and are being used extensively in the market in a wide range of products [248].

### 4.2. Benzalkonium Chlorides as Emerging Alternative Antimicrobials

Benzalkonium chlorides are the most widely used compounds currently deferred to as an eligible active ingredient for consumer antiseptic products in the final rule of the 2019 U.S. FDA Federal Register [248]. Due to the extensive use of BACs as cationic surfactants, phase transfer agents, fabric softeners, disinfectants, detergents, biocides and as ingredients in various personal care and pharmaceutical preparations, BACs have been detected in environmental samples of surface water, runoff water, sediment, wastewater and sewage sludge [254,255,256,258,259,260,261,262,263,264,265]. The most commonly used BACs are alkyldimethylbenzylammonium chlorides, in which the quaternary ammonium ion contains two methyl groups and one benzyl group on one side and a long-chain alkyl group (with typical even-numbered carbon atoms C_8_-C_18_) on the other side. BACs are a broad class of homologous *n*-alkylbenzyldimethylammonium chlorides with varying numbers of carbon atoms in the alkyl chain. Commercial formulations use either one or a mixture of homologues in order to meet the selective strength and activity of applications. The strength is influenced by the length of the alkyl chain [266]. The most commonly used commercial products contain BAC-12, BAC-14 and BAC-16, referring to 12, 14 and 16 carbon atoms in the alkyl chain, respectively [267,268]. The hydrophobic alkyl end plays a contributory role to the adsorption characteristics of these compounds and, hence, their fate, partitioning and distribution in the environment [254,269,270]. The physicochemical properties of commonly used BACs are shown in Table 5.

### 4.3. Degradation Byproducts/Metabolites of Benzalkonium Chlorides

The biodegradation pathways for BACs in wastewater and sludge have not yet been well established. Some research papers have shown degradation mechanisms along with their byproducts and metabolites in the wastewater treatment systems during chemical oxidation processes [273,274,275,276]. In water-treatment systems, the formation of disinfection byproducts (DBPs), particularly *N*-nitrosodimethylamine (NDMA), has been well studied due to the carcinogenic characteristics of NDMA [276,277,278,279,280,281]. BACs contain a quaternary nitrogen atom which can serve as a possible precursor for the formation of DBPs [282,283,284]. BACs are used as algaecides in swimming pools in combination with chlorination and UV-irradiation disinfection treatments. The cumulative process of disinfection proceeded to the formation of DBP intermediates through radical substitution reactions in the long alkyl chain of BACs. The primary products have been shown to serve as a source and precursor for the final DBPs of BACs [283].

**Figure 8:**
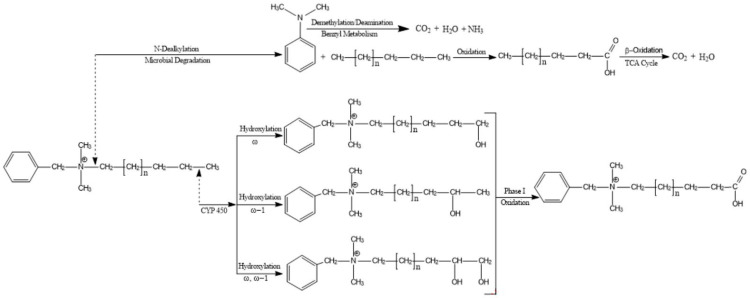
Benzalkonium chloride (*n* = 6, 8 and 10) metabolism scheme (adapted from references [285,288,290]).

Microbial biodegradation of BACs has been reported to produce benzyldimethylamine as the primary metabolite through the initiation of *N*-dealkylation at the active site of the quaternary nitrogen atom [285]. The alky intermediates in all instances were converted to carboxylic acids and the ultimate end products were obtained by the *β*-oxidation metabolic pathway through the citric acid cycle [285,286]. Bacterial metabolism of BACs by sewage and soil microorganisms suggested that the terminal carbon of the alkyl moiety is oxidized to carboxylic acids, with further oxidation to end products [286]. The amine intermediates provide the final end products of carbon dioxide, water and ammonia by the metabolic mechanisms of dealkylation and deamination [287,288,289]. The metabolism of BACs in the human body is key for its excretion, persistence and health effects. The un-metabolized portion of BACs in the blood stream will exert systemic toxicological effects. A recent study using human hepatic cytochrome P450 enzymes (CYP) responsible for xenobiotic and drug metabolism revealed that the metabolism takes place in the long-chain alkyl end, unlike *N*-dealkylation cleavage at the quaternary ammonium active site [290]. CYP-mediated alkyl chain oxidation at ω and ω-1 carbon positions rendered hydroxy, dihydroxy, ketone and carboxylic acid metabolites. The current literature-reported metabolic pathways are summarized in Figure 8.

### 4.4. Benzalkonium Chlorides in Sludge and Biosolids

BACs with positively-charged nitrogen atoms tend to accumulate in sludge due to their strong biosorption affinity [291,292]. BACs have been identified with a selected group of surfactants in treated and untreated sludge [293]. A summary of BACs found in sludge are presented in Table 6. Kümmerer et al. [294] reported that the annual discharge of BACs from different European hospitals to sewage effluents was approximately 1000 kg. The emitted amount ultimately accumulated in sludge and it was found that the total sum of BAC-12 and BAC-14 in the digested sludge of three different Swedish WWTPs was greater than 1000 kg/yr, highlighting potential concerns for the use of sludge for land application [257]. Furthermore, sorption kinetic studies of municipal sludge and lime-stabilized biosolids revealed that the sorption affinity of BACs is very high, while mobility in aqueous phase is very low [295,296]. The presence of the commonly encountered BAC-10, BAC-12, BAC-14 and BAC-16 in digested sludge provides evidence of the wide distribution of BAC homologues in sludge [261]. The total concentrations of BACs in Chinese sewage sludge were estimated in the range of 0.09–191 µg/g dry weight [256]. Sewage sludge-derived contamination has been considered to be the cause for the occurrence of BACs in soils and sediments [255,297,298]. The concentrations of BAC-12 and BAC-14 in the Taiwanese river Tam Sui were 2.1 and 0.8 µg/L, respectively [299]. In recent research, the average concentration of BAC-12 in wastewater influents and effluents in China was 1500 and 5.1 ng/L, respectively. The influent and effluent concentrations for BAC-14 were 586.7 and 2.5 ng/L, respectively [264]. The average concentrations of the downstream river water samples for BAC-12 and BAC-14 were 1.6 and 1.2 ng/L, respectively [264]. The biodegradation metabolites benzyl methyl amine and dodecyldimethyl amine of BACs were also identified from the returned activated sludge of a domestic WWTP in Ontario, Canada [289].

### 4.5. Fate, Transformation and Impacts of Benzalkonium Chlorides in the Environment

The ultimate sink of BACs is wastewater treatment systems through which they can enter into different environmental compartments [255,292,302]. The key transport mechanism is dissolution to the aqueous phase through cleaning and washing [263]. Cationic surfactants also contribute to the solubility and mobility of other co-existing compounds in the sewage [256]. In wastewater treatment processes, hydrophobic and electrical interactions with organic matter predominantly bind these compounds for physical separation [254,268,291,296]. The longer the alkyl chain, the higher the Log K_ow_ values and, hence, the binding affinities. The reported Log K_ow_ values for BAC-12, BAC-14 and BAC-16 are 2.93, 3.91 and 4.89, respectively [264,271]. The environmental fate and transformations are governed by a combination of physical, chemical and biological factors [255]. The diagenesis process also takes place in the sorbed fractions in the sludge and sediment over time [258,302]. Biodegradation and abiotic transformations are not significant for BACs [270,298]. Therefore, physical separation by sorption is an important contributor to the fate and environmental distribution of BACs [291,295,296,303].

Benzalkonium chlorides may have adverse impacts on aquatic organisms in the environment. BAC-12, BAC-14 and BAC-16 demonstrated acute 96-h EC50 toxicity values of 0.597, 0.473 and 0.406 µmol/L, respectively, on the microalgae *Chlorella vulgaris* [271]. Kreuzinger et al. [304] reported EC50 values of as low as 41 µg BAC/L (72-h) for algae (*Pseudokirchneriella subcapitata*), 125 µg BAC/L (48-h) for rotifers (*Brachionus calyciflorus*) and IC50 values as low as 41 µg BAC/L (48-h) for daphnids (*Daphnia magna*). The potential for these compounds to have an impact will likely increase as environmental concentrations rise due to limited removals during wastewater treatment and increased use of these compounds as manufactures shift away from legacy antimicrobials.

### 4.6. Future Challenges and Concerns around Benzalkonium Chlorides

The unique physical and chemical properties of BACs allow them to be used for a variety of applications and they have become popular in a large number of industrial and domestic applications. Due to the recent outbreak of the novel coronavirus, massive hand sanitization and sterilization led to a drastic rise in the market of BACs in order to stop the spread of the disease and to protect public health [252]. The health effects due to the excessive use of these high-production-volume chemicals are of grave concern. Some potential health risks associated with the use of these products include birth defects, fertility issues, skin irritation, allergies, cancer and genotoxicity [290,305,306,307,308]. Among the QACs, benzalkonium chlorides (BACs) are one of the major classes which possess antimicrobial properties and relatively high lethal dose (LD50) values in rats (Table 7). A recent report linked BACs in disinfectant products to developmental defects in mice, while other researchers have found that QACs can negatively impact cellular processes [309]. The purpose of the use of the disinfectants is to kill or inactivate harmful organisms. However, destroying or disturbing beneficial microorganisms in biological wastewater treatment processes cannot be avoided. Therefore, the potential risk goes beyond the intended boundary to human health and to the environment [310].

There are also increasing concerns regarding the development of antibiotic-resistant bacteria from the environmental release of BACs [288,312,313,314,315,316,317,318]. BACs are in the class of halogenated organic compounds that can be persistent in the environment [298]. The presence and wide distribution of BACs in aquatic water resources, sludge, sediment and agricultural land poses multiple risks to public health and to the environment [258,260,265,305,317,319,320,321,322,323]. Considering these risk factors and challenges, the U.S. FDA already issued a ban on one of the active quaternary ammonium ingredients, benzethonium chloride [248]. The antimicrobial ingredients of BACs may potentially be harmful, similar to banned ingredients [324]. Therefore, it is imperative that any potential risks from emerging antimicrobials are understood well-before their use becomes even more common and before these chemicals become even more widespread in the environment.

## 5. Conclusions

Legacy antimicrobials have been a fixture of modern life for over 40 years. Two of the most common legacy antimicrobials are TCS and TCC, which have toxic, persistent and bioaccumulative characteristics. These chemicals are widespread environmental contaminants due to large production volumes, heavy usage, limited degradation during wastewater treatment and their persistence in the environment. Although aqueous phase removals of up to 98% of TCS and 99% of TCC during wastewater treatment have been reported, the primary removal mechanism is partitioning to wastewater sludge. Although some TCS and TCC removals have been observed during various forms of biological sludge treatments (typically <40% during conventional anaerobic digestion), concentrations up to 19,000 ng/g dw of TCS and 63,000 ng/g dw of TCC have been reported. Furthermore, while a number of biotic and abiotic processes have been shown to be somewhat effective in degrading these chemicals, they often generate significant quantities of metabolites and byproducts, many of which may be even more toxic and environmentally persistent than their parent antimicrobials. Recent studies are increasingly suggesting that these chemicals and their byproducts may not be as innocuous as was first thought and may have adverse short- and long-term environmental and human health impacts.

Several emerging alternative antimicrobials, including various benzyl-substituted quaternary ammonium chlorides, are rapidly replacing legacy antimicrobials due to bans on active ingredients and shifting consumer preferences. There are growing concerns that many of these replacements and their byproducts present similar environmental and human health concerns as legacy antimicrobials. Few studies to date have adequately explored these alternatives so further work is urgently needed to ensure that legacy antimicrobials are not replaced by chemicals which may present similar or even possibly even greater risks.

## Figures and Tables

**Table 1:** Physicochemical properties of triclosan.

Property	Triclosan (TCS)
Formula	C_12_H_7_Cl_3_O_2_
CAS number	3380-34-5
Structure	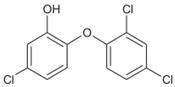
Molecular weight (g/mole)	289.5 g/mole
Log K_ow_	4.76 ^†^
Solubility in water (mg/L at 20 °C)	10.0 ^‡^
pKa	7.9 ^†^
Melting Point (°C)	55–57 ^‡^

CAS: Chemical abstract service; Log K_ow_: logarithmic octanol–water partition coefficient; pKa: dissociation constant. ^†^ https://ntp.niehs.nih.gov/ntp/htdocs/chem_background/exsumpdf/triclosan_508.pdf; ^‡^ https://pubchem.ncbi.nlm.nih.gov/compound/5564.

**Table 2:** Occurrences of triclosan and its transformation products/metabolites in sludge and biosolids.

Sludge Source	Treatment Conditions	TCS Levels in Raw Sludge	TCS Levels in Treated Sludge	Transformation Products/Metabolites Levels in Sludge	Reference
Three different WWTPs: WWTP (A): Primary treatment only (small facility <25,000 people). WWTP (B): Chemically enhanced primary treatment (large facility >1 million people). WWTP (C): Primary + secondary BNR treatment (medium facility >500,000 people).	WWTP (A): Aerobic digestion. WWTP (B): None. WWTP (C): MH anaerobic digestion.	WWTP (A): 2247 ± 185 ng/g dw WWTP (B): 447 ± 21 ng/g dw WWTP (C): 196 ± 14 ng/g dw (primary) and 174 ± 12 ng/g dw in secondary sludges	WWTP (A): 2299 ng/g dw WWTP (B): 393 ng/g dw WWTP (C): 310 ng/g dw	Study also examined TCS transformation products/metabolites: • 111 ng/g dw 2,4-DCP found in digested sludge from WWTP (B) (< LOD of 1.0 ng/g dw in WWTPs (A) and (B)) • MeTCS was 262 ng/g dw in digested sludge from WWTP (A); < LOD of 1.0 ng/g dw in WWTP (B) and 26.2 ng/g dw in WWTP (C) • DCDD was < LOD (3 ng/g dw) in all three facilities	[126]
Very large east-coast U.S. municipal WWTP (daily flow 1.25 million m^3^/day). Facility uses primary sedimentation, activated sludge and tertiary treatment (including nitrification–denitrification).	Cambi™ thermal hydrolysis followed by anaerobic digestion (TH-AD). Anaerobic digester operated at 37 °C with a 22-day SRT.	Mixed sludge before TH-AD treatment: TCS: 6884 to 7489 ng/g dw; MeTCS: 248 to 280 ng/g dw; 2,4-DCP: 204 to 283 ng/g dw.	10,872 to 12,345 ng/g dw	Study also examined TCS transformation products/metabolites. Effluent from TH-AD contained: • MeTCS: 326 to 340 ng/g dw • 2,4-DCP: 396–661 ng/g dw • TCS-SO_4_ was not detected in samples collected within the TH-AD process	[127]
Heat-treated biosolid pellets from a full-scale WWTP in the United Kingdom.	n/a	n/a	11,220 to 28,220 ng/g dw	Biosolid pellets had 35 to 69 ng/g dw of MeTCS	[128]
Five American WWTPs, including two activated sludge (treating wastwater from ~400,000 and 27,000 people) and three tricking filter (all treating wastewater from <3100 people).	Full-scale aerobic digestion and anaerobic digestion of mixed sludge. Process specifics not provided.	• Activated sludge facilities: - 8750 to 14,700 ng/g dw TCS in primary; - 900 to 4,200 ng/g dw TCS in secondary sludges. • Tricking filter facilities: - 7500 to 12,200 ng/g dw TCS in primary; - 7300 ng/g dw TCS in secondary sludges.	• Digested sludge values not reported in activated sludge facilities • 530 to 15,600 ng/g dw in tricking filter facilities	Study also examined MeTCS and higher-chlorinated TCS compounds: • MeTCS in activated sludge facilities: - 50 to 260 ng/g dw in primary - 310 to 1030 ng/g dw in secondary • MeTCS in tricking filter facilities: - 140 to 200 ng/g dw in primary sludge - 450 ng/g dw in secondary sludge • Low levels of higher-chlorinated TCS in digested tricking filter sludge: - < LOD to 110 ng/g dw of 4,5-dichloro-2-(2,4-dichloro-phenoxy)-phenol - < LOD to 100 ng/g dw of 5,6-dichloro-2-(2,4-dichloro-phenoxy)-phenol - <LOD and 80 ng/g dw of 4,5,6-trichloro-2- (2,4-dichloro-phenoxy)-phenol	[41]
Mixed fermented primary and secondary sludge (33:67% by weight) from a medium-sized Canadian BNR WWTP (>40,000 people).	• Bench-scale anaerobic digester (35 °C) and cycling aerobic/anoxic (22 °C) digesters operated at SRTs between 5 and 20 days • Sequential AD (35 °C) and cycling aerobic/anoxic (22 and 35 °C, respectively) digesters operated at various first: second stage SRTs (total SRT of 20 days).	Mixed sludge contained: • 2323 to 6127 ng/g dw TCS; • 111 to 214 ng/g dw TCS-SO_4_.	• Anaerobic digester effluent with 4832 to 7080 ng/g dw at different SRTs • Aerobic/anoxic digester effluent with 1183 and 4532 ng/g dw at different SRTs • Sequential digester effluent with 2430 to 6875 ng/g dw	Study also examined TCS transformation products/metabolites: • Low levels of 2,4-DCP were found in anaerobic digester effluent but were below LOQ. Anaerobic digesters were also effective in removing TCS-SO_4_, which had levels between 91.8 and 194.1 ng/g dw in effluent • 2,4-DCP below LOD in aerobic/anoxic digester effluent, near-complete removal of TCS-SO_4_ (15.0 to 40.1 ng/g dw in effluent) • Near complete removal of TCS-SO_4_ in sequential anaerobic/aerobic/anoxic digesters (11.3 and 137.6 ng/g dw)	[80,129]
Mixed fermented primary and secondary sludge (33:67% by volume) from a medium-sized Canadian BNR WWTP (>40,000 people).	Bench-scale anaerobic digesters operated at SRTs of 20, 12 and 6 days under MH and TH temperatures.	TCS in mixed primary and secondary BNR sludge: • 6760 to 8240 ng/g dw during cold months; • 1700 to 3530 ng/g dw during warmer months • Mean conc.: 4450 ± 2750 ng/g dw	• 8775 ng/g to 10,990 ng/g dw for TH digestates at different SRTs • 9120 to 12,790 ng/g dw for MH digestates at different SRTs	Study examined TCS-gluc, TCS-SO_4_, 5,6- dichloro-2-(2,4-dichloro-phenoxy)-phenol and 4,5,6-trichloro-2-(2,4-dichloro-phenoxy)-phenol and 2,3,4-TCP, but were below the LOD (1.44, 0.12, 0.58, 0.09 and 0.26 ppb, respectively)	[124]
19 full-scale Spanish WWTPs located in a mix of rural, industrial and urban areas.	n/a	n/a	54 to 2987 ng/g dw with a mean concentration of 1194 ng/g dw	4 to 311 ng/g dw for MeTCS, with a mean concentration of 54 ng/g dw	[130]
Large WWTP in the Mid-Atlantic region of the U.S. employing primary treatment and secondary treatment (activated sludge with added nitrification–denitrification).	Dewatered and treated with lime (~15% by weight).	n/a	19,100 ng/g dw	1000 ng/g dw MeTCS	[131,132]
Large WWTP in the Mid-Atlantic region of the U.S. employing primary treatment and secondary treatment (activated sludge with added nitrification–denitrification).	Dewatered and treated with lime (~15% by weight).	• 19,200 ng/g dw TCS • 80 ng/g dw MeTCS	16,200 ng/g dw TCS	160 ± 30 ng/g dw MeTCS	[42]

TCS: triclosan; WWTP: wastewater treatment plant; BNR: biological nutrient removal; MeTCS: methyl triclosan; dw: dry weight; n/a: data not avaiable; LOD: limit of detection; 2,4-DCP: 2,4-dichlorophenol; 2,3,4-TCP: 2,3,4-trichlorophenol; DCDD: dichlorodibenzo-p-dioxins; TH-AD: Cambi™ thermal hydrolysis followed by anaerobic digestion; TCS-SO_4_: triclosan sulfate; TCS-gluc: glucuronidated triclosan; SRTs: solids retention times; MH: mesophilic; TH: thermophilic; LOQ: limit of quantification.

**Table 3:** Key physicochemical properties of triclocarban.

Property	Triclocarban (TCC)
Formula	C_13_H_9_Cl_3_N_2_O
CAS number	101-20-2
Structure	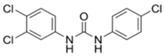
Molecular weight (g/mole)	315.578
Log K_ow_ (at 25 °C, pH 7)	4.9 ^†^
Solubility in water (mg/L at 25 °C)	0.65–1.55 ^†^
pKa	12.7 ^‡^
Vapour pressure (mm Hg at 25 °C)	3.61 × 10^−9 §^
Melting point (°C)	255–256 ^‡^

CAS: Chemical abstract service; Log K_ow_: logarithmic octanol–water partition coefficient; pKa: dissociation constant. ^†^ Halden and Paull [176]; ^‡^ Wu et al. [144]; ^§^ Ying et al. [143].

**Table 4:** Occurrences of triclocarban and its transformation products/metabolites in sludge and biosolids.

Sludge Source	Treatment Conditions	TCC Levels in Raw Sludge	TCC Levels in Treated Sludge	Transformation Products/Metabolites Levels in Sludge	Reference
Primary sludge samples from various WWTPs located in five different U.S. states	n/a	7500 to 25,900 ng/g dw for primary sludge, with a mean concentration of 19,300 ± 7100 ng/g dw	n/a	• 3′-Cl-TCC: 80 to 500 ng/g dw for primary sludge, with a mean concentration of 275 ± 150 ng/g dw • DCC: 7 to 30 ng/g dw for primary sludge, with a mean concentration of 16 ± 9 ng/g dw	[213]
100 sewage sludge samples from 25 full-scale WWTPs in 18 U.S. states	n/a	n/a	•21,600 to 43,200 ng/g dw for undigested sludge^†^ (median conc.: 34,900 ± 7200 ng/g dw) •4700 to 63,000 ng/g dw for anaerobically digested biosolids (median conc.: 27,600 ± 9600 ng/g dw) •16,400 to 19,600 ng/g dw for aerobically digested biosolids (median conc.: 18,000 ng/g dw)	• 3′-Cl-TCC: - 1700 to 4300 ng/g dw for undigested sludge - 40 to 5000 ng/g dw for anaerobically digested biosolids - 2400 to 2700 ng/g dw for aerobically digested biosolids • DCC: - 250 to 590 ng/g dw for undigested sludge - 30 to 14,900 ng/g dw for anaerobically digested biosolids - 210 to 740 ng/g dw for aerobically digested biosolids	[201]
100 sewage sludge samples from 86 domestic and 16 industrial WWTPs in China	n/a	<3 to 43,300 ng/g dw with 95% DF	n/a	• 3′-Cl-TCC: 22 to 580 ng/g dw with 100% DF • DCC: <2 to 23,890 ng/g dw with 94% DF • MCC: <2 to 120 ng/g dw with 92% DF • NCC: 3 to 1340 ng/g dw with 90% DF • 2′−OH-TCC: <1 to 2340 ng/g dw with 94% DF • 3′−OH-TCC: <1 to 1250 ng/g dw with 91% DF	[206]
Un-pretreated and MW-pretreated mixed sludge samples (FPS:TWAS = 33:67% by volume)	Bench-scale anaerobic digesters operated at SRTs of 20, 12 and 6 days under MH and TH temperatures	300 to 1800 ng/g dw for mixed sludge with a mean concentration of 1030 ± 470 ng/g dw	• 620 to 1900 ng/g dw for TH digestates at different SRTs • 940 to 1900 ng/g dw for MH digestates at different SRTs	• NCC: 250 to 1020 ng/g dw for TH digestates and 130 to 675 ng/g dw for MH digestates at different SRTs • 4-Cl-TCC, monochloroanilines and 4-chlorocatechol: below the LOD (0.70, 0.12 and 0.87 ppb, respectively) • MCC and DCC: below the LOQ for digestates (0.25 and 0.25 ppb, respectively)	[44]

TCC: triclocarban; WWTP: wastewater treatment plant; n/a: data not available; DF: detection frequencies; 3′-Cl-TCC: 3,3′,4,4′-tetrachlorocarbanilide; DCC: dichlorocarbanilide; MCC: monocarbanilide; NCC: carbanilide; 2′−OH-TCC: 2′-hydroxy-triclocarban, 3′−OH-TCC: 3′-hydroxy-triclocarban, FPS: fermented primary sludge; TWAS: thickened waste activated sludge; MW: microwave; SRTs: solids retention times; MH: mesophilic; TH: thermophilic; LOD: limit of detection; LOQ: limit of quantification. ^†^ includes different types of sludge treatment, such as thickening or lime stabilization.

**Table 5:** Physicochemical properties of benzalkonium chlorides.

Property	Benzalkonium Compounds		
	BAC-12	BAC-14	BAC-16
Formula	C_21_H_38_ClN	C_23_H_42_ClN	C_25_H_46_ClN
CAS number	139-07-1	139-08-2	122-18-9
Structure	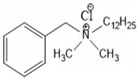	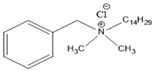	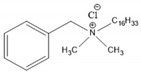
Molecular weight (g/mole)	339.99 g/mole	368.04 g/mole	396.09 g/mole
Log K_ow_	2.93 ^†^	3.91 ^†^	4.89 ^‡^
Solubility in water (mg/L at 25 °C)	1230 ^§^	100 ^§^	8.5 ^§^
pKa	NA	NA	NA
Melting point (°C)	42–44 *	47–52 *	54–56 *

CAS: Chemical abstract services; Log K_ow_: logarithmic octanol-water partition coefficient; pKa: dissociation constant; NA: not applicable; BAC-12/BAC-14/BAC-16: benzalkonium chlorides containing 12, 14 and 16 carbon atoms in the alkyl chain, respectively. ^†^ W.-L Li et al. [264]; ^‡^ M. Zhu et al., [271]; ^§^ P. Daull et al., [272]; * https://www.trc-canada.com/product-detail/.

**Table 6:** Occurrence of benzalkonium chlorides in sludge and biosolids.

Sludge Source	Treatment Conditions	BAC Levels in Raw Sludge	BAC Levels in Treated Sludge	Transformation Products/Metabolites in Sludge	Reference
Treated, untreated and digested sludge samples from 11 Swedish WWTPs	• Full-scale biological process in different treatment combinations • 10 WWTPs used aerobic sludge digestion while only one WWTP did not have sludge digestion	n/a	• BAC-10: 24 to 210 ng/g dw for digested sludge (median conc.: 100 ng/g dw) • BAC-12: 8800 to 89,000 ng/g dw for digested sludge (median conc.: 29,000 ng/g dw) • BAC-14: 3200 to 60,000 ng/g dw for digested sludge (median conc.: 17,000 ng/g dw) • BAC-16: 990 to 4900 ng/g dw for digested sludge (median conc.: 2200 ng/g dw)	n/a	[261]
Spiked sludge used to check adsorption by wastewater sludge	Bench-scale unit that consisted of a Pyrex glass tube was used for thermocatalytic low temperature conversion of sludge	• BAC-12: 0.1 mg/g dw spiked dewatered sludge • BAC-14: 0.1 mg/g dw spiked dewatered sludge	• BAC-12: 0.1 mg/g spiked dried sludge, *n* = 9 • BAC-14: 0.1 mg/g spiked dried sludge, *n* = 3	n/a	[300]
Digested sludge samples from three different Swedish WWTPs	Full-scale conventional biological process of different sizes and treatment configurations • Plant 1: Secondary clarifier sludge treated by anaerobic digesters at temperature of 34–37 °C and SRT of 15 days • Plant 2: Primary clarifier sludge of 1.7-h retention time introduced to the digester with SRT of 20 days at 37 °C Plant 3: Primary sludge introduced to the digesters, treated with SRT of 25–30 days at 37 °C	• BAC-12: The total influent mass flow percentage of BAC-12 for primary sludge in the three WWTPs: 62, 55 and 25%, respectively • BAC-14: The total influent mass flow percentage of BAC-14 for primary sludge in the three WWTPs: 74, 54 and 41%, respectively	• BAC-12: Total influent mass flow percentage of BAC-12 for digested sludge in the three WWTPs: 67, 43 and 42%, respectively • BAC-14: The influent mass flow percentage of BAC-14 for digested sludge at the three WWTPS: 96, 65 and 74%, respectively	n/a	[257]
Sewage sludge samples from 52 municipalities in China	Full-scale conventional biological treatment process of various combinations for municipal WWTPs	n/a	• BAC-8 to BAC-18 homologues: 0.09 to 190 µg/g dw for freshly digested freeze-dried sludge (median conc.: 1.09 µg/g dw)	n/a	[256]
Anaerobic digestion of waste activated sludge collected from a local WWTP in Harbin, China	Full-scale conventional activated sludge process for treating municipal wastewater	• BACs: The base level of BACs in raw sludge was 48 ± 7 µg/g TSS	• BAC: Waste-activated sludge was amended with BACs at 2, 8, 15, 25 and 50 mg/g of TSS to investigate performance and microbial community responses of anaerobic digestion	n/a	[301]

BACs: benzalkonium chlorides; BAC-8/BAC-10/BAC-12/BAC-14/BAC-16/BAC-18: benzalkonium chlorides containing 8, 10, 12, 14, 16 and 18 carbon atoms in the alkyl chain, respectively; dw: dry weight; SRT: solids retention time; WWTP: wastewater treatment plant; n/a: data not available; n: number of measurements; TSS: total suspended solids.

**Table 7:** Lethal dose (LD50) for benzalkonium chloride in rats.

Exposure Route	LD50 Concentration
Intravenous	14 mg/kg body weight
Oral	240 mg/kg body weight
Intraperitoneal	15 mg/kg body weight
Subcutaneous	400 mg/kg body weight

Adapted from reference [311].

## Abbreviations

2,4-DCP	2,4-dichlorophenol
2,3,4-TCP	2,3,4-trichlorophenol
3,4-DCA	3,4-dichloroaniline
4-CA	4-chloroaniline
4-CC	4-chlorocatechol
4-Cl-TCC	3,3′,4,4′-tetrachlorocarbanilide
BACs	Benzalkonium chlorides
BAC-8	Benzalkonium chlorides containing eight carbon atoms in the alkyl chain
BAC-10	Benzalkonium chlorides containing ten carbon atoms in the alkyl chain
BAC-12	Benzalkonium chlorides containing twelve carbon atoms in the alkyl chain
BAC-14	Benzalkonium chlorides containing fourteen carbon atoms in the alkyl chain
BAC-16	Benzalkonium chlorides containing sixteen carbon atoms in the alkyl chain
BAC-18	Benzalkonium chlorides containing eighteen carbon atoms in the alkyl chain
BNR	Biological nutrient removal
CAS	Chemical abstract service
CYP	Cytochrome P450 enzymes
DBPs	Disinfection byproducts
DCC	Dichlorocarbanilide
DCDDs	Dichlorodibenzo-*p*-dioxins
DL	Detection limit
dw	Dry weight
*E. coli*	*Escherichia coli*
IC25	25% inhibition concentration
LC50	Median lethal concentration
LD50	Lethal dose at which 50 percent of the population cannot survive
Log K_ow_	Logarithmic octanol-water partitioning coefficients
MCC	Monochlorocarbanilide
MeTCS	Methyl triclosan
MH	Mesophilic
MRSA	Methicillin-resistant *Staphylococcus aureus*
NCC	Carbanilide
NDMA	*N*-Nitrosodimethylamine
NR	Not reported
OLR	Organic loading rate
p*K*a	Dissociation constant
PPCPs	Pharmaceuticals and personal care products
QACs	Quaternary ammonium compounds
SRT	Sludge retention time/solids retention time
TCC	Triclocarban
TCS	Triclosan
TCS-gluc	Glucuronidated triclosan
TCS-SO_4_	Triclosan sulfate
TH	Thermophilic
TH-AD	Cambi Thermal Hydrolysis followed by anaerobic digestion
U.S.	United States
U.S. EPA	United States Environmental Protecion Agency
U.S. FDA	United States Food and Drug Administration
UV	Ultraviolet
WWTPs	Wastewater treatment plants
